# Integrating systematic biological and proteomics strategies to explore the pharmacological mechanism of danshen yin modified on atherosclerosis

**DOI:** 10.1111/jcmm.15979

**Published:** 2020-11-02

**Authors:** Kailin Yang, Liuting Zeng, Anqi Ge, Xiaoping Pan, Tingting Bao, Zhiyong Long, Qiaozhen Tong, Mengxia Yuan, Xiaofei Zhu, Jinwen Ge, Zhengde Huang

**Affiliations:** ^1^ The First Affiliated Hospital of Hunan University of Chinese Medicine Changsha China; ^2^ Hunan University of Chinese Medicine Changsha China; ^3^ Capital Medical University Beijing China; ^4^ Department of Rheumatology and Clinical Immunology Peking Union Medical College Hospital, Chinese Academy of Medical Sciences & Peking Union Medical College Beijing China; ^5^ Guang'anmen Hospital, China Academy of Chinese Medical Sciences Beijing China; ^6^ Beijing University of Chinese Medicine Beijing China; ^7^ Shantou University Medical College Shantou China; ^8^ Xiangya School of Medicine Central South University Changsha City China

**Keywords:** ApoE‐/‐ mouse, atherosclerosis, Danshen Yin Modified, proteomics, reverse transcription‐PCR, systematic biology

## Abstract

This research utilized the systematic biological and proteomics strategies to explore the regulatory mechanism of Danshen Yin Modified (DSYM) on atherosclerosis (AS) biological network. The traditional Chinese medicine database and HPLC was used to find the active compounds of DSYM, Pharmmapper database was used to predict potential targets, and OMIM database and GeneCards database were used to collect AS targets. String database was utilized to obtain the other protein of proteomics proteins and the protein‐protein interaction (PPI) data of DSYM targets, AS genes, proteomics proteins and other proteins. The Cytoscape 3.7.1 software was utilized to construct and analyse the network. The DAVID database is used to discover the biological processes and signalling pathways that these proteins aggregate. Finally, animal experiments and proteomics analysis were used to further verify the prediction results. The results showed that 140 active compounds, 405 DSYM targets and 590 AS genes were obtained, and 51 differentially expressed proteins were identified in the DSYM‐treated ApoE‐/‐ mouse AS model. A total of 4 major networks and a number of their derivative networks were constructed and analysed. The prediction results showed that DSYM can regulate AS‐related biological processes and signalling pathways. Animal experiments have also shown that DSYM has a therapeutic effect on ApoE‐/‐mouse AS model (*P* < .05). Therefore, this study proposed a new method based on systems biology, proteomics, and experimental pharmacology, and analysed the pharmacological mechanism of DSYM. DSYM may achieve therapeutic effects by regulating AS‐related signalling pathways and biological processes found in this research.

## 1 INTRODUCTION

Atherosclerosis (AS) is the most common disease in arteriosclerosis, mainly involving the elastic arteries and the more muscular fibres of the elastic fibres. The typical atherosclerotic lesion contains a large amount of ‘ather‐like’ components of lipids and necrotic cells.^1^ AS is a major disease that seriously endangers human health. The main clinical events of death are coronary heart disease and stroke. Cardiovascular and cerebrovascular diseases are the leading causes of death worldwide.^1^, ^2^ In 2008, more than 17 million people worldwide died of cardiovascular and cerebrovascular diseases, of which 7.3 million died of coronary heart disease and 6.2 million died of stroke. It is estimated that by 2030, the number of deaths from cardiovascular and cerebrovascular diseases worldwide will reach 23.3 million.^1^, ^2^, ^3^

Currently, it is believed that atherosclerotic disease is the result of a variety of complex factors interacting.^4^ The current study found that it is mainly related to lipid metabolism disorders, endothelial damage, inflammatory response, wall shear stress and intestinal microflora imbalance.^5^, ^6^ The therapeutic drugs for AS include hypolipidemic drugs, antiplatelet drugs, dilated vascular drugs and treatment for coronary heart disease caused by ischaemia.^7^, ^8^, ^9^ However, as these drugs require patients to take their medications for life, their side effects result in low patient compliance and reduced quality of life.^10^, ^11^

In addition, the treatment of AS‐related cardiovascular and cerebrovascular diseases has entered a stage of diversified comprehensive treatment. Complementary and alternative medicine (CAM) has gradually entered mainstream medicine and has become a popular choice for patients.^12^ Traditional Chinese Medicine (TCM), as an important component of CAM, has accumulated thousands of years of horizontal and vertical clinical experience in the treatment of AS. Among them, Danshen Yin (DSY) is from the Shi Ge Fang Kuo, which has the effect of promoting blood circulation and relieving pain in Chinese medicine theory. As a classic prescription, it has been treating coronary heart disease and angina as its establishment.^13^ Our previous clinical studies ^13^, ^14^ and other reports ^15^ showed that DSY Modified (DSYM) can reduce the total myocardial ischaemic load in patients with unstable angina (low‐risk group, intermediate‐risk group) and has anti‐ischaemic effects. Its specific mechanism may be related to lowering serum myocardial enzyme level, reducing myocardial infarct size, improving myocardial cell ultrastructure and inhibiting cardiomyocyte apoptosis.^16^ Our previous work also showed that the mechanism of DSYM intervention in coronary heart disease may be related to autophagy and oxidative stress.^17^, ^18^ More importantly, previous researches have only studied single signal pathways or a few targets; and it is not easy to reveal the synergistic effect of ‘multi‐component‐multi‐target’ herbs and the regulation effects of herbs on biological network of the disease from a holistic and comprehensive perspective. However, the mechanism of DSYM intervention in coronary heart disease has not been elaborated, especially its role in AS, the basic lesions of coronary heart disease. Our previous research successfully used systematic biological methods (such as network pharmacology and systematic pharmacology) to explore the mechanism of herbal formulae for treating diseases.^19^, ^20^, ^21^, ^22^, ^23^ Hence, this study will explore the mechanism of DSYM regulating AS’s biological network by an integrated systematic biological and proteomics strategies, and provide new ideas for drug development. The research processes were shown in Figure S1.

## 2 MATERIALS AND METHODS

### 2.1 Data preparation for systems biological analysis

#### 2.1.1 DSYM’s potential components prediction

With ‘*Radix Paeoniae Rubra*’, ‘*Chuanxiong Rhizoma*’, ‘*Carthami Flos*’, ‘*Rehmanniae Radix Praeparata*’, ‘*Radix Salviae*’, ‘*Angelicae Sinensis Radix*’ and ‘*Santalum Album L*.’ as the key word, all the components of DSYM with their pharmacokinetic parameters [oral bioavailability (OB), Caco‐2 permeability and drug‐likeness (DL)] ^24^, ^25^, ^26^, ^27^ were searched in TCM Database@Taiwan ^28^ (http://tcm.cmu.edu.tw/zh‐tw/) and the TCMSP (http://lsp.nwsuaf.edu.cn).^29^ The compounds with OB ≥ 30%, Caco‐2> −0.4 and DL ≥ 0.18 were considered to be orally absorbable and pharmacologically active (namely, potential compounds).^19^, ^20^, ^21^, ^22^, ^23^, ^24^, ^25^, ^26^, ^27^, ^29^

Due to the limitation of predicting potential components based on pharmacokinetic parameters only,^30^ in order to avoid omission of components, we searched a large number of literatures to supplement orally absorbable compounds with bioactive. Finally, after searching, a total of 34 components were added from the reference.^31^, ^32^, ^33^, ^34^, ^35^, ^36^, ^37^, ^38^, ^39^, ^40^, ^41^, ^42^

#### 2.1.2 DSYM’s potential targets prediction and as genes collection

The molecular structures of DSYM’s potential components were collected from the SciFinder (http://scifinder.cas.org) and PubChem (https://pubchem.ncbi.nlm.nih.gov/), and were drawn by ChemBioDraw and saved as ‘mol2’ file format. Then, they were input into PharmMapper (http://lilab‐ecust.cn/pharmmapper) to predict the potential targets of DSYM.^43^ The UniProtKB (http://www.uniprot.org/) was utilized to collect the official symbols of the potential target's proteins with the species limited to ‘Homo sapiens’. (see Table S1). Meanwhile, OMIM database (http://omim.org/) and Genecards (http://www.genecards.org) were utilized to collect the AS‐related disease genes and targets.^44^, ^45^ The AS‐related genes and their relevance scores are shown in Table S2.

### 2.2 Network Construction and Analysis Methods

The protein‐protein interaction (PPI) data were obtained from the String database (http://string‐db.org/).^46^ The networks were constructed by Cytoscape 3.7.1 software (https://cytoscape.org/), a software to graphically display, analyse and edit the network.^47^ Degree refers to the number of connections to the node. Betweenness refers to the number of edges passing through the nodes. Degree and betweenness can determine the importance of the topology of the nodes in the network. The larger the value, the more important it is.^47^ The networks were further analysed by the plug‐in of Cytoscape, MCODE to found the clusters.^48^ The definition and the methodology of acquisition of clusters were described in our previous work.^19^, ^20^, ^21^, ^22^, ^23^

### 2.3 Gene ontology (GO) and pathway enrichment analysis

The genes and targets in clusters were input into DAVID (https://david‐d.ncifcrf.gov, ver. 6.8) to perform GO enrichment analysis. All of the genes and targets in the networks were also input into DAVID for Kyoto Encyclopedia of Genes and Genomes (KEGG) signalling pathway enrichment analysis.^48^ *P*‐value is Modified Fisher Exact P‐Value, EASE score.^48^ The smaller, the more enriched.

### 2.4 Experimental materials

#### 2.4.1 Experimental animal

Fifty specific‐pathogen‐free (SPF) degree 8‐week‐old apolipoprotein E gene knockout (ApoE‐/‐) male mice (Bodyweight 20‐22g) are provided by Beijing Weitong Lihua Experimental Animal Technology Co., Ltd. (Animal certificate number: SCXK(京)2012‐0001); Twelve SPF 8‐week‐old C57BL/6L male mice (Bodyweight 20‐22g) are provided by Hunan Slack Jingda Experimental Animal Co., Ltd. (Animal certificate number: SCXK(湘)2011‐0003). Animals are housed in IVC‐ II isolation cage with food and ultrapure water provided ad libitum and maintained at temperature of 20‐25°C with 55 ± 5% relative humidity, pressure difference 20‐50 Pa and a 12 h light/dark cycle. All experiments were conducted in accordance with Chinese national laws and local guidelines. All animals' care and experimental procedures were approved by the Animal Ethics Committee of Hunan University of Chinese Medicine and were in accordance with the National Institute of Health's Guide for the Care and Use of Laboratory Animals. And the experiment is complied with the ARRIVE guidelines.

#### 2.4.2 Laboratory apparatus

High‐speed refrigerated centrifuge Allegra 25R (Beckman Inc), Radioimmunoassay ac1500r (China University of Science and Technology), UV/Vis spectrophotometer Lambda 35 (Perkin Elmar Inc, USA), Ultra‐thin slicer LKBIII (Pharmacia LKB Biotechnology, Swedish), Transmission electron microscope, Tecnaig2 Spirit (FEI Company), Electronic balance GB204 (Mitler Inc, Switzerland), Constant temperature water bath DK‐SD (Shanghai Jinghong Experimental Equipment Co., Ltd.), Ice machine AF100As (Scotsman Inc, Italy), Ultrapure water machine SIMSV0000 (Millipare Inc), Automatic Biochemical Analyzer CL‐7200 (Roche Group), Chemiluminescence E2010 (Roche Group), Microplate reader 318C+ (Shanghai Peiou Analytical Instrument Co., Ltd.), Independent air supply isolation cage type IVC‐II (Suzhou Fengshi Experimental Animal Equipment Co., Ltd.), Medical purification workbench CJ‐2F (Suzhou Fengshi Experimental Animal Equipment Co., Ltd.), Stainless steel steaming decanter model ZLSC‐5 (Shanghai Shenan Instrument Factory), Electrophoresis instrument DYY‐2C (Beijing Liuyi Instrument Factory), Vertical plate electrophoresis instrument DYCZ‐24DN (Beijing Liuyi Instrument Factory), Transfer electrophoresis tank type VE186 (Shanghai Tianneng Technology Co., Ltd.), Horizontal shaker WD‐9405B (Beijing Liuyi Instrument Factory), Ultra pure water machine SIMSV0000 (Merck Millipore Inc, USA), PCR machine MASTERCYCLER (Eppendorf Inc, Germany), Gel Imaging System Tanon 16 008 (Shanghai Tianneng Technology Co., Ltd.), StepOne Real‐Time PCR System (Life Technolo gies), Fluorescent Scanner Scanning Instrument Genepix (Molecular Devices Inc, USA), RayBio QAM‐CYT‐4000 Protein Chip Analysis Software (Ray Biotech Inc, USA). Agilent 1200 High Performance Liquid Chromatograph (HPLC) (UV‐VIS Detector, Solution Chromatography Workstation); American Phenomenex C18 Column (4.6mm × 250 mm, 5 μm); HEMGDING FA 2004 Electronic Analytical Balance (Shanghai Hengping Scientific Instrument Co., Ltd., d = 0.1 mg); KQ‐500DE type numerical control ultrasonic cleaner (Kunshan Ultrasonic Instrument Co., Ltd.).

#### 2.4.3 Laboratory reagent

Nitric Oxide (NO), Endothelin (ET) Kit (Nanjing Institute of Bioengineering; Catalog No.: A012‐01‐02, B013‐01‐83); Mouse hs‐CRP (chemiluminescence) kit (Monobind Inc, USA; Catalog No.: 8.02774.0100); VEGF, MMP‐9 Enzyme Linked Immunoassay Kit (Xiamen Huijia Biotechnology Co., Ltd.; Catalog No.: E1097‐A442, T1096‐A012); Cholesterol (Changsha Lixin Biological Co., Ltd.); Simvastatin tablets (production batch number: K010176; Catalog No.: 02013697) (Hangzhou Merck Pharmaceutical Co., Ltd.); Aspirin enteric‐coated tablets (production batch number: BJ19147; Catalog No.: 11014828) (Bayer Health Care Co., Ltd.). Reverse Transcription Kit (Fcrnientans Inc, USA; Catalog No.: K0171); QAM‐CAA‐4000 Antibody Protein Chip Kit (Ray Biotech Inc, USA; Catalog No.: 43513). Tanshinone IIA (batch number: 111539‐201212; Catalog No.: 008135; purity: 99.82%), salvianolic acid B (batch number: 111517‐201212; Catalog No.: 111362; purity: 99.76%), tanshinol (batch number: 2154‐201211; Catalog No.: 110855; purity: 99.65%), rosmarinic acid (batch number: 111628‐201211; Catalog No.: 111871; purity: 99.21%), protocatechuic aldehyde (batch number: 111813‐201211; Catalog No.: 110810; purity: 99.01%) (China Food and Drug Control Institute). Anhydrous methanol (99.7%, analytical grade), formic acid (analytical grade), acetonitrile (TEDIA, chromatographically pure) (Zhongguo Group Chemical Reagent Co., Ltd.); re‐distilled water. Chinese herbal medicine (): *Radix Salviae* (batch number: 405375L; Catalog No.: 90230261), *Santalum Album L*. (batch number: 311086L; Catalog No.: 9079031), *Radix Paeoniae Rubra* (batch number: 404217L; Catalog No.: 9079056), *Chuanxiong Rhizoma* (batch number: 404109L; Catalog No.: 9079024),*Angelicae Sinensis Radix* (batch number: 404371L; Catalog No.: 9012146), *Carthami Flos* (batch number: 405239L; Catalog No.: 9014089), *Rehmanniae Radix Praeparata*. (batch number: 404070L; Catalog No.: 9078028) were purchased from the Pharmacy of the First Affiliated Hospital of Hunan University of Chinese Medicine (produced by Guangdong Yifang Pharmaceutical Co., Ltd.) and were appraised by Professor Zhou Ribao from the Department of Traditional Chinese Medicine Resources, Hunan University of Chinese Medicine.

### 2.5 Experiment method

#### 2.5.1 Drug preparation

Danshenyin Modefied formula (DSYM): This formula consists of *Radix Salviae* 20g, *Santalum Album L*. 6g, *Angelicae Sinensis Radix* 6g, *Rehmanniae Radix Praeparata* 12g, *Chuanxiong Rhizoma* 6g, *Radix Paeoniae Rubra* 10g and *Carthami Flos* 6g. Those herbs were mixed and soaked for 3 minutes, and boiled 2 times (add *Santalum Album L*. after 10‐20 minutes after boiling start). The decoctions were combined, filtered and concentrated to 1 g of original medicinal material/ mL. Finally, they were stored at 4°C.

Simvastatin + aspirin suspension: The tablets are dissolved in distilled water and concentrated to the solution containing simvastatin 0.2 mg/ mL and aspirin 1 mg/ mL.

#### 2.5.2 Animal models, grouping and drug administration

Twelve (12) C57BL/6L mice were set to blank group and fed with normal diet. Fifty (50) male ApoE (‐/‐) mice were fed a standard Western diet (containing 0.15% cholesterol and 21% fat wt/wt).^49^ After 12 weeks of modelling, they were randomly divided into model group, simvastatin + aspirin control group, DSYM low dose group, and DSYM high dose group. Among them, DSYM low dose group and DSYM high dose group contained 13 male mice per group, while the remaining groups contained 12 male mice per group.

After the successful modelling, the drug intervention was started. The dose was calculated according to the weight ratio of 60 kg human and 30 g mouse: the control group was administered with simvastatin at 2.52 mg/(kg·d) and aspirin at 12.60 mg/(kg·d); DSYM low dose group was administered with a solution containing 8.31 g/kg/d of crude drug; DSYM high dose group was administered with a solution containing 24.93 g/kg/d of crude drug. The blank group and the model group were given equal doses of ultrapure water per day according to the bodyweight ratio. The bodyweight was weighed once a week to adjust the dose, and the intervention was continued for 8 weeks.

#### 2.5.3 Specimen preparation method

After 0.5 h of the last administration, 2% sodium pentobarbital solution was prepared, and the mice were anaesthetized by intraperitoneal injection at a dose of 180 mg/kg. After the anaesthesia was effective, the abdominal cavity of the mouse was opened, and 1.5 mL of blood was taken through the abdominal aorta and allowed to stand for 30 minutes. The blood samples were centrifuged for 10 minutes at 4°C, 3 000 r/min within 1 hour and the supernatant was aspirated with a pipette, stored in a dry EP tube at −20°C for the detection of blood lipids, NO, ET, hs‐CRP, VEGF, MMP‐9, etc The mice were killed by cervical dislocation, and a small section of aortic specimen connected to the heart was quickly taken out about 1‐2 cm under sterile conditions. Some specimens were fixed with 4% paraformaldehyde for HE staining, some were fixed by 2.5% glutaraldehyde for electron microscopy, and some were stored in liquid nitrogen for Western Blot, RT‐PCR, etc

#### 2.5.4 Determination of Serum NO and ET

The NO is determined by the nitrate reductase method and is performed according to the instructions of the NO kits. Absorbance values of each tube were measured using a 550 nm wavelength and a 0.5 cm path. Calculate the NO concentration according to the formula. The experiment was repeated three times, and the average value was taken.

Calculation formula: NO concentration (μmol/L) = (measurement tube absorbance ‐ blank tube absorbance)/ (standard tube absorbance ‐ blank tube absorbance) * standard tube concentration * sample dilution factor.

The ET was measured by radioimmunoassay and was administered by the Department of Nuclear Medicine of the First Affiliated Hospital of Hunan University of Chinese Medicine. The experiment was performed according to the instructions of the ET kits. The radioactivity of the precipitate was measured on an automatic gamma counter, and the concentration of ET was calculated from the standard curve, and the result was automatically obtained by a computer. The experiment was repeated three times, and the average value was taken.

#### 2.5.5 Determination of blood lipid

Serum total cholesterol (TC), triglyceride (TG), low‐density lipoprotein (LDL‐C) and high‐density lipoprotein (HDL‐C) levels were measured by an automatic biochemical analyzer. It is responsible by the biochemical room of the First Affiliated Hospital of Hunan University of Chinese Medicine. The experiment was repeated three times, and the average value was taken.

#### 2.5.6 Determination of serum hs‐CRP

The serum hs‐CRP level was determined by chemiluminescence method. It is responsible by the biochemical immunization room of the First Affiliated Hospital of Hunan University of Chinese Medicine. The experiment was repeated three times, and the average value was taken.

#### 2.5.7 Determination of serum VEGF and MMP‐9

The serum VEGF and MMP‐9 levels were determined by double‐antibody sandwich enzyme‐linked immunosorbent assay (ELISA) and performed in strict accordance with the instructions in the kit. Allow the kit to warm to room temperature for at least 20 minutes before use. All reagents and samples are configured in advance. The sample is provided with 3 duplicate holes. Blank wells, standard wells and sample wells were set; 100 μL of the sample dilution was added to the blank wells, and 100 μL of the standard or the sample was added to the remaining wells. The plate was coated with a cover and incubated at 37°C for 120 minutes; after the incubation is complete, the liquid is discarded. Then, 100 μL of biotin‐labelled antibody working solution was added to each well and incubated at 37°C for 60 min; after the incubation, the liquid was discarded and the plate was washed. The horseradish peroxidase labelled avidin working solution (with biotinylated antibody working solution) 100ul was then added into each well and incubated at 37°C for 60 minutes; after the incubation, the liquid was discarded and the plate was washed. After that, the substrate solution was added into each well the colour was developed at 37°C in the dark for 30 minutes. Finally, the reaction was stopped by adding 50 ul of the stop solution to each well (the blue colour turned yellow at this time); and the optical density (OD value) of each well was measured sequentially at 450 nm using an enzyme‐linked instrument (The reference wavelength is 630 nm).

#### 2.5.8 Morphological changes of aortic roots in mice observed by HE staining

The specimen was fixed with 4% paraformaldehyde, dehydrated with an upstream gradient, and paraffin embedded after hyalinized by xylene. The specimen was continuously cross‐sectioned from the heart to the ascending aorta 5 μm up to the aortic root (100 μm above the aortic valve).^50^ Approximately 20 slices were taken in succession, and one was taken every 5 sheets. Then, HE staining is performed. Under the X100, X204 and X400 light microscope, it was observed that whether the intima of the vessel wall is bulged, whether there is accumulation of foam cells under the intima, whether the internal elastic membrane is intact, and photographed.

#### 2.5.9 Ultrastructural observation of aortic endothelial cells

One (1) mm^3^ aortic root tissue in the mice of each group were taken out and fixed with 2.5% glutaraldehyde phosphate buffer for 2 hours or longer; Then, the tissue was rinsed with 0.1 mol/L phosphoric acid rinsing solution and fixed with 1% citrate fixative for 1 ~ 2 h. After dehydration, soaking, embedding, and curing, the specimen was cut into thin slices of 50‐100 nm (70 nm) by LKB‐III ultrathin microtome, and double‐stained with 3% uranyl acetate and lead nitrate. Finally, FEI Tecnai G2 Spirit transmission electron microscope was used to observe and film.

### 2.6 Protein chip detection method for dsym intervention in ApoE‐/‐ mice aortic tissue

#### 2.6.1 Tissue protein extraction

The frozen tissue was taken out from the liquid nitrogen, and lysis solution was added at a ratio of 500 ul of lysis solution per 0.1 mg of tissue. The tissue was broken up on a high‐speed dispersing cutter on ice. Centrifuge at 12 000 rpm for 20 minutes in a refrigerated centrifuge and take the supernatant.

#### 2.6.2 Complete drying of the slide chip

Remove the slide chip and recombine at room temperature for 20‐30 min. Open the package, uncover the seal and place the chip in a vacuum desiccator or dry at room temperature for 1‐2 h.

#### 2.6.3 Chip operation

This process is carried out in strict accordance with the instructions in the QAM‐CAA‐4000 Antibody Protein Chip Kit: 100 μL of sample dilution was added to each well (final protein concentration of 500 μg/mL), and it was incubated for 30 minutes at room temperature on a shaker to block the quantitative antibody chip. After incubation, the buffer in each well was removed, 100 μl of standard and sample (diluted to 500 μg/mL) was added to the wells and incubated overnight at 4°C on the shaker. Then, 1.4 mL of the sample dilution was added to antibody mixture tubules, mixed well and the tubules were centrifuged; after that, 80 μL of detection antibody was added to each well and incubated for 2 hours on a shaker at room temperature. Then, 1.4 mL of the sample dilution was added to Cy3‐streptavidin tubules, mixed well and the tubules were centrifuged; after that, 80 μL of Cy3‐streptavidin was added to each well and incubated for 2 hours on a shaker in the dark. Finally, the Axon genePix were utilized to scan the signal using Cy3 or a green channel. The scanning parameters are as follows: PMT: 600, Wavelength: 532 nm, resolution: 10 μm. The data analysis was performed using QAM‐CAA‐4000 data analysis software.

### 2.7 Validation of VEGF, MMP‐9 and bFGF by RT‐PCR

Aortic tissue samples were taken, and total RNA was extracted as described in the instructions of Trizol kit. After dilution with 2 μL of RNA, the RNA purity and concentration were determined by UV spectrophotometry. Using total RNA as a template, cDNA was synthesized according to the instructions of the reverse transcription kit, and the obtained DNA was amplified in a 25 μL system. The PCR amplification conditions were as follows: pre‐denaturation at 95°C for 5 minutes, 95°C for 10 seconds, 60°C for 1 minutes, and 40 cycles. The Ct value was read after the reaction was completed. The specificity of the PCR reaction of each sample was monitored by melting curve, and β‐actin was used as an internal reference gene for data analysis. The primers were shown in Table 1. The experiment was repeated three times, and the average value was taken.

**Table 1:** Primers of VEGF, MMP9, bFGF and actin

Primers	Forward primer	Reverse primer	Product length
VEGF	5'‐AACGATGAAGCCCTGGAGT‐3'	5'‐CATCTGCTGTGCTGTAGGAAG‐3'	122 bp
MMP9	5'‐ GCGfiCGTGATCCCCACTTAC‐3'	5'‐CAGGCCGAA'T'AGGAGCGTC‐3'	88 bp
bFGF	5'‐GCGACCCACACGTCAAACTA‐3'	5'‐CCGTCCATCTTCCTTCATAGC‐3'	104 bp
Actin	5‐CCACCATGTACCCAGGCATT‐3'	5'‐CGGACTCATCGTACTCCTGC‐3'	189 bp

### 2.8 Determination of compounds in DSYM using HPLC

#### 2.8.1 HPLC conditions

Column Phenomenex C18 column (4.6 mm × 250 mm, 5 μm); Mobile phase: methanol (A)—formic acid (B)—acetonitrile (C), gradient elution: 0 ~ 10min (10%A‐80%B‐10%C), 10 ~ 20 min (30%A‐60%B‐10%C), 20 ~ 30 min(50%A‐40%B‐10%C), 30 ~ 45min (70%A‐20%B‐10%C), 45 ~ 50 min (80%A‐10%B‐10%C), 50 ~ 55 min (10%A‐80%B‐10%C); Detection wavelength: 270 nm; column temperature: 30°C; flow rate: 1 mL/min; injection volume: 10 μL. The number of theoretical plates should be ≥ 2 000 according to the peak of tanshinone II A.

#### 2.8.2 DSYM sample preparation

According to the DSYM prescription, 66.28 g of dry herbal pieces was accurately weighed and placed in a 500 mL flat‐bottomed flask. The mixture was condensed and refluxed with 10 times and 8 times of water for 1 hour, filtered, and the filtrate was combined. The volume of filtrate was adjusted to 1000ml with distilled water, and the filtrate was filtered through a 0.45 μm PTEF microporous filter membrane, shaved with tin foil and placed in the refrigerator for later use.

#### 2.8.3 Standard sample preparation

According to the method in reference,^51^ the tanshinone IIA, salvianolic acid B, tanshinol, rosmarinic acid, protocatechuic aldehyde were accurately weigh and distilled water were added to prepare a mixed solution per ml containing 160 μg of tanshinone IIA, 140 μg of salvianolic acid B, 16 μg of tanshinol, 10 μg of protocatechuic aldehyde and 23 μg of rosmarinic acid.

### 2.9 Statistical analysis

All data were processed using SPSS 22.0 statistical software. One‐way analysis of variance was used for comparison of multiple groups, and the measurement data were expressed as mean ± standard deviation. The measurement data were measured by mean ± standard deviation. The test level *P* < .05 indicated that the difference was statistically significant.

## 3 RESULTS AND DISCUSSION

### 3.1 Atherosclerosis PPI network analysis

#### 3.1.1 Atherosclerosis PPI network

**Figure 1:**
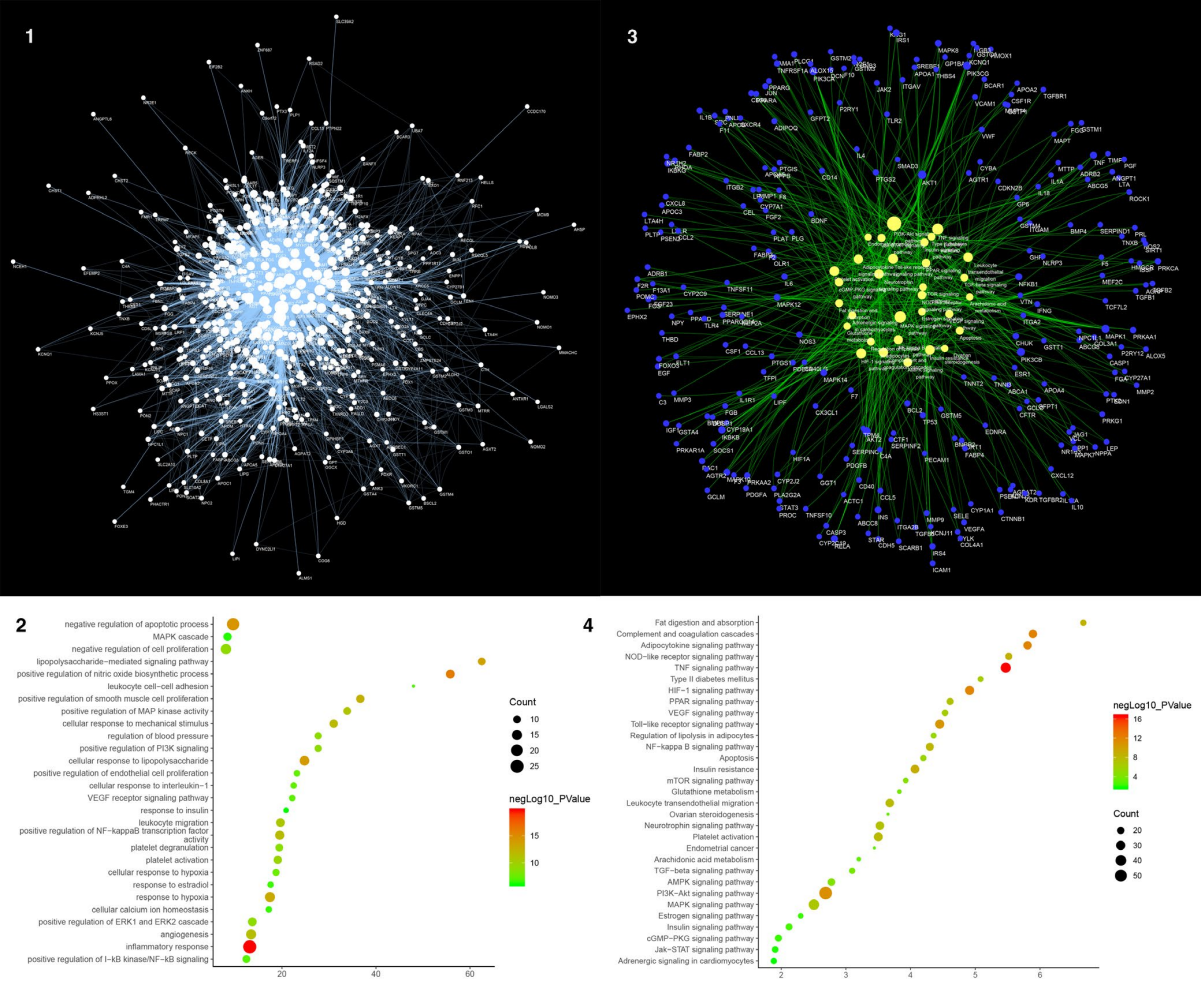
Result of AS PPI Network Analysis. A, Atherosclerosis PPI Network (The larger the node size, the higher the degree of the node. The thicker the line, the greater the Edge Betweenness of the node.). B, Bubble chart of biological processes (X‐axis is fold enrichment). C, Pathway of AS Network (Blue circle stands for AS genes. Yellow circle stands for signalling pathway. The larger the node size, the higher the degree of the node. The thicker the line, the greater the Edge Betweenness of the node.). D, Bubble Chart of Signalling Pathway (X‐axis is fold enrichment)

Three thousand and seven hundred and twenty‐nine (3729) AS‐related genes were required from GeneCards and OMIM database. The PPI data of 590 genes whose relevance score ≥ 3 were obtained to construct the AS PPI network, and this network contains 531 nodes and 11 853 edges (Figure 1A). These genes are arranged in descending order of the relevance score, the top 10 are as follows: APOE (Score: 63.76), APOB (Score: 50.94), APOA1 (Score: 47.42), LDLR (Score: 46.84), ABCA1 (Score: 40.75), CETP (Score: 37.14), LMNA (Score: 34.7), ABCG8 (Score: 33.38), PPARG (Score: 33.31) and LPL (Score: 32.28).

The genes in the network are arranged in descending order of degree. The top 10 are as follows: ALB (294 edges), INS (262 edges), IL6 (235 edges), VEGFA (227 edges), TNF (226 edges), AKT1 (219 edges), TP53 (198 edges), JUN (190 edges), EGF (181 edges) and PIK3CA (174 edges) (Figure 1A). Meanwhile, the genes with high relevance score also have high degrees: APOE (174 edges), APOB (119 edges), APOA1 (92 edges), LDLR (99 edges), ABCA1 (68 edges), CETP (39 edges), LMNA (18 edges), ABCG8 (32 edges), PPARG (123 edges) and LPL (85 edges).

#### 3.1.2 Biological Processes of AS PPI Network

The AS PPI network was analysed by MCODE and returns 18 clusters (Table 2 and Figure S2). The genes in the clusters were input into DAVID for GO enrichment analysis.

**Table 2:** Cluster of AS network

Cluster	Score	Nodes	Edges	Genes
1	64.602	84	2681	PLG, ESR1, REN, TP53, NOS3, AKT1, IL6, INS, PIK3CA, TNF, FOXP3, ACE, KDR, VCAM1, PIK3CG, VEGFA, MMP9, SERPINE1, ICAM1, NOTCH1, TLR4, IFNG, SELP, CDKN2A, EDN1, EGF, NFKB1, CSF1, IGF1, MAPK1, LEP, IL4, CXCL12, ALB, F2, MMP2, IL17A, CASP1, CCL5, CAT, IL10, F3, TNFSF11, JUN, RELA, ITGAM, IL1B, BDNF, RHOA, HMOX1, SPP1, MAPK14, PTGS2, CAV1, STAT3, ANXA5, MMP1, FOS, CD34, NR3C1, TIMP1, CXCR4, BCL2, JAK2, TGFB1, MAPK8, CD40, EGR1, TNFRSF1A, SRC, TNFSF10, APOE, LDLR, CD40LG, GFAP, CTNNB1, PIK3CB, FGF2, KNG1, PPARG, CASP3, CCL2, ITGA2, SELE
2	10.133	16	76	MTHFR, CYP2C19, GSTT1, ALOX5, GSTO1, GSTA4, GSTM1, PLA2G2A, CYP2C9, GSTM3, GSTM2, GSTM4, GSTM5, DNMT1, CYP3A5, GSTP1
3	9.943	36	174	VCL, LCAT, APOA2, LPA, SDC2, HMGCR, JAG1, C3, APOC2, SREBF2, APOA5, APOA4, AGTR2, F2R, EDNRA, PLTP, ITGB2, TGFBR1, PSEN1, ITGAV, ABCG1, NPC1L1, UTS2, NR1H3, APOA1, APOC1, ABCA1, CETP, MTTP, HTR2A, NOTCH4, SERPINC1, LIPC, P2RY1, ABCG5, CYP7A1
4	9.889	37	178	F5, CHUK, SERPINF2, F8, ELANE, DUSP1, BMP4, FGB, NCF1, FGG, IL1R1, CXCR2, PPARA, ANGPT1, CFTR, PPBP, CCL11, AHSG, TXN, HMGB1, ITGA2B, ITIH4, TGFB3, CLU, CD36, MMP14, ENG, APOB, APOH, VTN, TGFB2, DCN, CDH5, FGA, PTK2, F13A1, PF4
5	8.875	33	142	AGTR1, AGT, CCR2, CCR5, PGF, FOXO3, ADIPOQ, SIRT1, IL1A, SMAD3, FLT1, NOS2, PLAT, SCARB1, SOCS1, CSF1R, TLR2, HIF1A, ACTA2, ELN, SOD2, VWF, IGFBP3, MMP3, APP, IRS1, SELL, PRL, CYBB, MPO, CRP, IL18, IKBKB
6	6	6	15	XRCC5, WRN, NBN, FEN1, RFC1, EXO1
7	5.111	10	23	MTR, TXNRD2, BHMT, AHCY, SLC10A2, CBS, MTRR, ABCC2, NR1I2, CTH
8	4.875	17	39	BCAR1, P2RY12, SREBF1, ITGB3, CYP19A1, NPY, CXCL16, SST, GNB3, PRKCA, IGFBP1, LPL, RETN, F7, PDGFB, APLN, CASR
9	4	5	8	SQSTM1, NQO1, GCLC, GCLM, KEAP1
10	4	4	6	BMP6, BMPR2, TGFBR3, ACVRL1
11	4	4	6	RAN, MGP, NDE1, SERPINB6
12	3.429	22	36	PCSK9, ADM, PROC, F10, PRKAA2, VIP, TFPI, HP, SERPIND1, CP, COG2, TTR, NR1H4, COX5A, ABCG8, NAMPT, PPARGC1A, PRKAA1, GHRL, MME, SHBG, UCP2
13	3	3	3	GPIHBP1, APOC3, ANGPTL3
14	3	3	3	SOAT1, SOAT2, CYP27A1
15	3	3	3	PDE5A, PRKAR1A, GP1BA
16	3	3	3	ALPL, COL4A1, IBSP
17	3	3	3	DYNC2LI1, COG6, ANK3
18	2.667	4	4	NFE2L2, HSPD1, SOD1, MAPK7

Cluster 1 is mainly involved in inflammation, lipid metabolism, oxidative stress, NO metabolism and so on. Cluster2 is associated with oxidative stress such as glutathione. Cluster 3 is mainly related to lipid metabolism and macrophage transformation to foam cells. Cluster 4 is primarily involved in the coagulation pathway and inflammatory response. Cluster 5 is primarily involved in hypoxia, inflammatory response, angiogenesis and lipid metabolism. Cluster 8 is associated with proliferation, migration of vascular endothelial cells and coagulation pathways. Cluster 12 is associated with lipid metabolism and coagulation pathways. Cluster 13 is related to lipid metabolism. Cluster 14 is mainly related to lipid metabolism and macrophage transformation to foam cells. Cluster 15 is related to coagulation pathways. Cluster 6, 7, 9, 10, 11, 16, 17 and 18 do not return AS‐related biological processes (Table S3). The main biological processes in cluster 1 were used as an example shown in Figure 1B.

#### 3.1.3 Pathways of AS Network

After the pathway enrichment analysis, thirty‐one AS‐related signalling pathways are obtained (Figure 1C). The P values, fold enrichment, and count of these signalling pathways were shown in Figure 1D. The details are described in Table S4.

Based on this network, it is found that ALB, INS, IL6, VEGFA, TNF, AKT1, TP53, JUN, EGF and PIK3CA are located at the core of the AS PPI network. Meanwhile, the genes with high relevance score (APOE, APOB, APOA1, LDLR, ABCA1, CETP, LMNA, ABCG8, PPARG and LPL) are also at an important position on the network. These genes may be targets for drug development in the future. TNF signalling pathway, Complement and coagulation cascades, Adipocytokine signalling pathway, HIF‐1 signalling pathway, PI3K‐Akt signalling pathway, Toll‐like receptor signalling pathway, Insulin resistance, NOD‐like receptor signalling pathway, Fat digestion and absorption and NF‐kappa B signalling pathway are the top 10 signalling pathways in the network, which may be the core pathways in the development of AS. In the network analysis, we also performed a GO enrichment analysis on the clusters of the network; and we found that AS‐related biological processes are mainly concentrated in the inflammatory module, cell modules such as vascular endothelial cells, smooth muscle cells and inflammatory cells, lipid metabolism modules, coagulation and fibrinolysis modules and so on.

Current research reports have shown that plaque instability in AS is the main cause of acute coronary syndrome (ACS). The pathological features of vulnerable plaque include thin fibrous cap, large lipid core, inflammatory cell infiltration, platelet aggregation, calcified nodules and vascular positive remodelling.^52^, ^53^, ^54^ High‐throughput omics revealed that biological processes affecting AS progression include cholesterol metabolism, proliferation and migration of vascular smooth muscle cells (VSMC), changes in macrophage function and foam cell formation, and intravascular calcification.^55^, ^56^, ^57^, ^58^ These pathophysiological processes interact, ultimately leading to endothelial dysfunction, lipid metabolism disorders, inflammation, oxidative stress and hemodynamic changes.

Various signalling molecules such as growth factors, cytokines, hormones and neurotransmitters regulate the lipid metabolism, inflammation and vascular endothelial function of AS through signalling pathways.^59^, ^60^ MAPK may mediate inflammatory processes, endothelial cell activation, smooth muscle cell proliferation, T lymphocyte differentiation, macrophage chemotaxis, foam cell formation, autophagy, apoptosis and activation, all of which are involved in the pathogenesis of AS.^61^, ^62^ In human smooth muscle cells, macrophages, and endothelial cells of the intimal/mediator and atherosclerotic regions of AS lesions, activation of NF⁃κB has been detected.^63^ The formation of foam cells is a key event in early AS, and NF⁃κB is inevitably involved. In promoting inflammation, NF⁃κB enhances the overexpression of important inflammatory factors and adhesion molecules. For example, TNF⁃α and IL⁃1β are induced by NF⁃κB, which also activates the NF⁃κB signalling pathway.^64^, ^65^ In addition, NF⁃κB plays an important role in aggravating arterial calcification, promoting platelet formation and rupture, and activating endothelial cells.^66^

TGF⁃β is a multifunctional cytokine involved in the inflammation and pathogenesis of AS, mainly involved in several key aspects of inflammation, chemotaxis, fibrosis, proliferation and apoptosis.^67^ Recent studies have shown that TGF⁃β may play a dual role in AS; in the early stages of AS, TGF⁃β acts to inhibit inflammation and increase the stability of AS plaques and inhibit T‐cell responses ^68^, ^69^; however, this effect will lost in the late stage due to changes in receptor expression.^70^

Endothelial insufficiency is the basis of cardiovascular events and an important feature of AS. A variety of cytokines and microRNAs inhibit endothelial cell survival by reducing the expression of STAT3 and inhibiting protein levels.^71^, ^72^, ^73^ In addition, down‐regulation of STAT3 can increase AS acute phase response and macrophage infiltration.^74^ Through the study of Toll‐like receptors (TLRs) pathway, it is found that AS is not only a metabolic disease, but also an immune inflammatory disease involving innate and adaptive immunity, which can cause aggravation of cardiovascular diseases such as myocardial infarction and stroke.^75^ As plaque stability is determined by inflammation and apoptosis, activation of the TLR pathway also accelerates plaque rupture.^76^

### 3.2 Compound‐compound targets network of DSYM

#### 3.2.1 Potential compounds of DSYM

After the potential compound protection and exceptional molecules collection, totally 137 potential compounds are collected. In these compounds, 103 are predicted by TCMSP: paeoniflorgenone, paeoniflorin, baicalein, beta‐sitosterol, sitosterol, Spinasterol, Stigmasterol, (+)‐catechin, (2R,3R)‐4‐methoxyl‐distylin (MOL006992), 1‐o‐beta‐d‐glucopyranosyl‐8‐o‐benzoylpaeonisuffrone (MOL006994), 1‐o‐beta‐d‐glucopyranosylpaeonisuffrone (MOL006996), stigmast‐7‐en‐3‐ol (MOL006999), Albiflorin, 4‐ethyl‐paeoniflorin (MOL007008), 4‐o‐methyl‐paeoniflorin (MOL007012), Paeoniflorigenone, 9‐ethyl‐neo‐paeoniaflorin A (MOL007018), evofolinB, Ethyl oleate (NF), campest‐5‐en‐3beta‐ol (474‐62‐4), Mandenol, Myricanone, Perlolyrine, senkyunone, wallichilide, 1,2,5,6‐tetrahydrotanshinone, Poriferasterol, poriferast‐5‐en‐3beta‐ol, isoimperatorin, sugiol, Dehydrotanshinone IIA, luteolin, α‐amyrin, 5,6‐dihydroxy‐7‐isopropyl‐1,1‐dimethyl‐2,3‐dihydrophenanthren‐4‐one (MOL007036), 2‐isopropyl‐8‐methylphenanthrene‐3,4‐dione (87112‐49‐0), 3α‐hydroxytanshinone IIA, (E)‐3‐[2‐(3,4‐dihydroxyphenyl)‐7‐hydroxy‐benzofuran‐4‐yl]acrylic acid (MOL007048), 4‐methylenemiltirone, 2‐(4‐hydroxy‐3‐methoxyphenyl)‐5‐(3‐hydroxypropyl)‐7‐methoxy‐3‐benzofurancarboxaldehyde (MOL007050), formyltanshinone, 3‐beta‐Hydroxymethyllenetanshiquinone, Methylenetanshinquinone, przewalskin A, przewalskin B, Przewaquinone B, przewaquinone C, (6S,7R)‐6,7‐dihydroxy‐1,6‐dimethyl‐8,9‐dihydro‐7H‐naphtho[8,7‐g]benzofuran‐10,11‐dione (MOL007070), przewaquinone F, sclareol, tanshinaldehyde, Danshenol B, Danshenol A, Salvilenone, cryptotanshinone, dan‐shexinkum D, danshenspiroketallactone, deoxyneocryptotanshinone, dihydrotanshinlactone, dihydrotanshinone I, epidanshenspiroketallactone, C09092, isocryptotanshi‐none, Isotanshinone II, manool, microstegiol, miltionone I, miltionone II, miltipolone, Miltirone, miltirone II, neocryptotanshinone II, neocryptotanshinone, 1‐methyl‐8,9‐dihydro‐7H‐naphtho[5,6‐g]benzofuran‐6,10,11‐trione (97399‐70‐7), prolithospermic acid, (2R)‐3‐(3,4‐dihydroxyphenyl)‐2‐[(Z)‐3‐(3,4‐dihydroxyphenyl)acryloyl]oxy‐propionic acid (537‐15‐5), (Z)‐3‐[2‐[(E)‐2‐(3,4‐dihydroxyphenyl)vinyl]‐3,4‐dihydroxyphenyl]acrylic acid (MOL007140), salvianolic acid g, salvilenone I, salviolone, NSC 122421, (6S)‐6‐hydroxy‐1‐methyl‐6‐methylol‐8,9‐dihydro‐7H‐naphtho[8,7‐g]benzofuran‐10,11‐quinone (97411‐46‐6), Tanshindiol B, Przewaquinone E, tanshinone IIA, (6S)‐6‐(hydroxymethyl)‐1,6‐dimethyl‐8,9‐dihydro‐7H‐naphtho[8,7‐g]benzofuran‐10,11‐dione (MOL007155), tanshinone VI, Flavoxanthin, 4‐[(E)‐4‐(3,5‐dimethoxy‐4‐oxo‐1‐cyclohexa‐2,5‐dienylidene)but‐2‐enylidene]‐2,6‐dimethoxycyclohexa‐2,5‐dien‐1‐one (MOL002694), lignan, lupeol‐palmitate, Phytoene, phytofluene, Pyrethrin II, 6‐Hydroxykaempferol, carthamone, 6‐Hydroxynaringenin, quercetagetin, 7,8‐dimethyl‐1H‐pyrimido[5,6‐g]quinoxaline‐2,4‐dione (MOL002757), beta‐carotene, kaempferol, CLR, quercetin, isorhamnetin.

Thirty‐four (34) compounds are collected from reference^31^, ^32^, ^33^, ^34^, ^35^, ^36^, ^37^, ^38^, ^39^, ^40^, ^41^, ^42^: Chlorogenic acid, ligustrazine, ferulic acid, senkyunolide I, senkyunolide H, senkyunolide A, butylidenephthalide, ligustilide, butylphthalide, catalpol, rehmannioside A, rehmannioside D, acteoside, stachyose, 5‐hydroxymethylfurfural, 6‐Hydroxykaempferol, kaempferol‐3‐O‐rutinoside, safflomin A, safflomin B, bidenoside C, gallic acid, protocatechuic acid, protocatechuic aldehyde, paeoniflorin sulphonate, methyl gallate, oxypaeoniflorin, ethyl gallate, benzoic acid, pentagalloylglucose, benzoyl‐paeoniflorin, paeonol, Santalol, vitexin and isovitexin.

#### 3.2.2 DSYM fingerprint

After comparing with the retention time of the standard and the spectrum, the five chemical components contained in the DSYM were determined: tanshinone IIA, salvianolic acid B, tanshinol, rosmarinic acid and protocatechuic aldehyde (Figure S3).

#### 3.2.3 Network construction

Based on the prediction of pharmacokinetic parameters, the supplement of literature and the results of HPLC, 140 components were introduced into pharmmapper for target prediction. This network is constructed by 581 nodes (405 compound target nodes and 140 compound nodes) and 31447 edges. The relationship between the targets and the compounds could be observed through the network. For example, INSR is regulated by Przewalskin A, 6‐Hydroxykaempferol, Benzoyl‐paeoniflorin, Danshenol B, Kaempferol‐3‐O‐rutinoside, MOL007018, Pyrethrin II, 6‐Hydroxynaringenin, Pentagalloylglucose, Senkyunolide H, Carthamone, MOL007050, Miltionone II, Prolithospermic acid and so on; F2 is regulated by all compounds. Paeonol can regulate BACE1, CA2, CCNA2, CDK2, F2, LCK, AKR1B1, FKBP1A, GSTP1, PDE4D, PTPN1, GSTA1, HSP90AA1, MMP3, GSK3B, HSD17B1, MAPK14, ABO, METAP2, AR, PRKACA, CHEK1, KDR, MAPK10, REN, ADK, CASP3, CTSK, SULT2B1, DHODH, HSD11B1, SRC, AKR1C3, KIF11, TGM3, MET, HMGCR, SULT2A1, ELANE, F10, FGFR1, MAOB, BCAT2, CYP2C9 and so on. Ligustrazine can regulate ADAM17, ADH, ADK, AKR1B1, AKR1C1, AKR1C2, AKR1C3, ALB, AMY1A, AMY1B, AMY1C, APOA2, AR, ARG1, BACE1, BCHE and so on (Figure S4).

### 3.3 DSYM‐AS PPI network analysis

#### 3.3.1 DSYM‐AS PPI network

**Figure 2:**
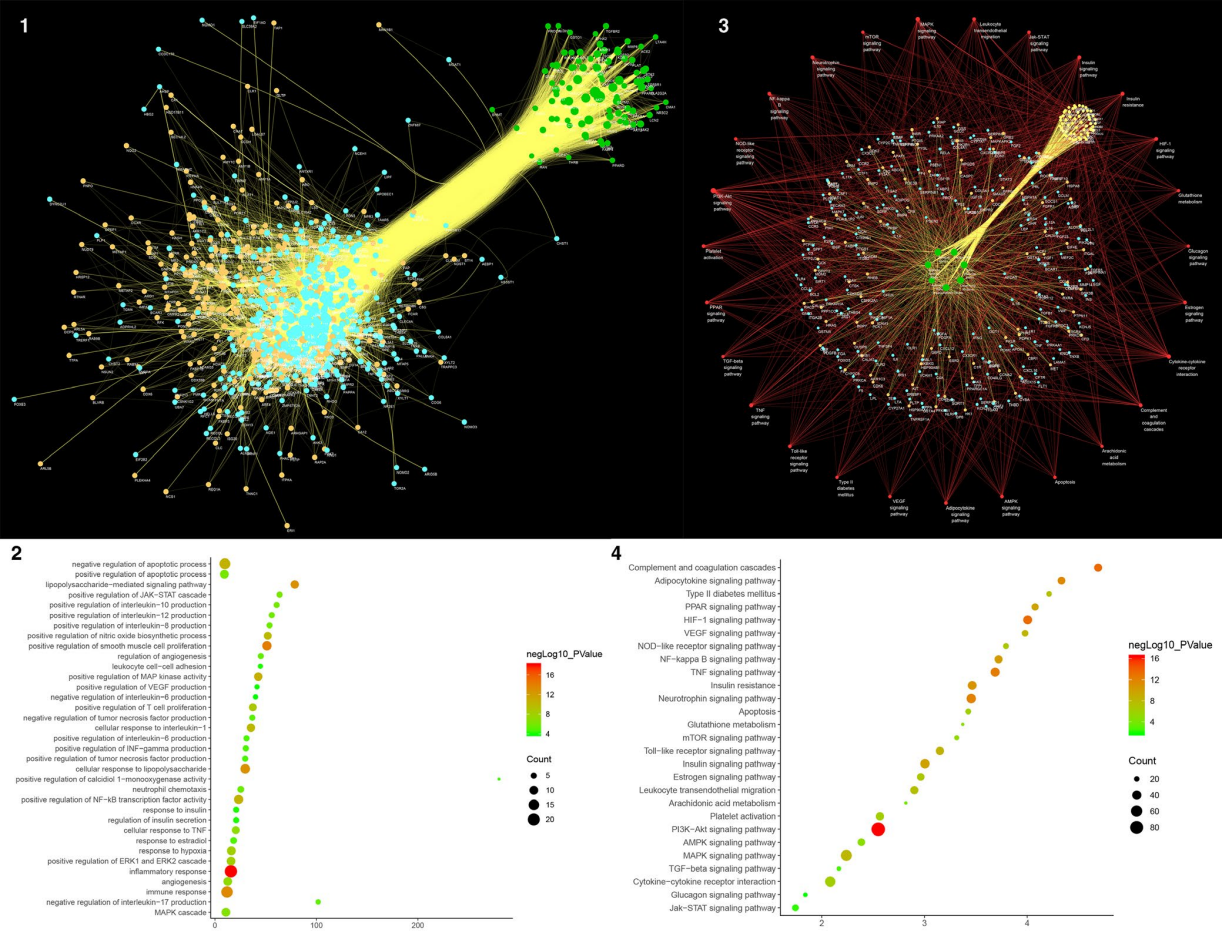
Results of DSYM‐AS PPI Network A, DSYM‐AS PPI Network (Blue, orange, green circles stand for AS genes, DSYM targets, and DSYM‐AS targets. The larger the node size, the higher the degree of the node. The thicker the line, the greater the Edge Betweenness of the node.). B, Bubble chart of biological processes (X‐axis is fold enrichment analysis). C, Pathway of DSYM‐AS PPI Network (Green circle stands for Herb. Red circle stands for signalling pathways. Blue circle stands for genes. Orange circle stands for DSYM targets. Yellow circle stands for DSYM‐AS targets. Red links stand for relationship among pathways and targets; yellow links stand for relationship among herbs and targets. The larger the node size, the higher the degree of the node. The thicker the line, the greater the Edge Betweenness of the node.). D, Bubble Chart of Signalling Pathway (X‐axis is fold enrichment analysis)

The DSYM‐AS PPI network was constructed based on DSYM targets, AS genes and their PPI data. This network contains 879 nodes (445 AS gene nodes, 342 DSYM target nodes and 92 DSYM‐AS targets nodes) and 24154 edges (Figure 2A). The top 20 targets of degree are selected and divided into three categories: (1) DSYM targets: EGFR (261 edges); (2) AS genes: INS (421 edges), IL6 (363 edges), TNF (330 edges), VEGFA (318 edges), TP53 (305 edges), EGF (263 edges), CXCL8 (237 edges), STAT3 (219 edges), IL1B (218 edges), JUN (215 edges), IL10 (215 edges); (3) DSYM‐AS targets: ALB (400 edges), AKT1 (357 edges), MAPK1 (250 edges), SRC (245 edges), MMP9 (243 edges), IGF1 (236 edges), CASP3 (233 edges) and MAPK8 (226 edges).

#### 3.3.2 Biological Processes of DSYM‐AS PPI Network

The DSYM‐AS PPI network was analysed by MCODE and returns 18 clusters (Table 3; Figure S5). The genes in the clusters were input into DAVID for GO enrichment analysis.

**Table 3:** Cluster of DSYM‐AS PPI network

Cluster	Score	Nodes	Edges	Genes and Targets
1	48.203	60	1422	BCL2L1, MMP3, MMP2, MPO, IL17A, CCL5, IL10, IL18, ELANE, ITGAM, JUN, IL1B, TNFSF11, RELA, BDNF, HMOX1, SPP1, LOX, PTGS2, IL2, STAT1, MMP1, STAT3, FOS, PECAM1, CD34, HIF1A, MMP13, TIMP1, TGFB1, CD40, MAPK8, TNFRSF1A, CXCL8, CD40LG, SRC, CTNNB1, CASP3, PPARG, FGF2, CCL2, PLG, TP53, IL6, TNF, VCAM1, ADIPOQ, FOXP3, MMP9, ICAM1, TLR4, IFNG, EDN1, MAPK1, CSF1, LEP, TLR2, IL4, MMP7, KIT
2	33.431	103	1705	AR, SERPINC1, RETN, CAT, CASP1, F3, CX3CR1, AGTR1, LGALS3, AGT, CST3, CAV1, IGF1R, SMAD3, MAP2K1, PGR, BMP2, TNFRSF11B, EGFR, ADAM17, FBN1, CSF1R, PTPN11, TGFB3, IL1RN, CFD, PLAU, TGFB2, CDH5, F13A1, PF4, REN, MDM2, AKT1, SERPINF2, FGB, CD163, KDR, NOTCH1, SIRT1, GPT, FGF23, NOS2, CDKN2A, NFKB1, HMGB1, CXCL12, TEK, SERPIND1, APP, SELL, GRB2, CCR5, CX3CL1, PROC, RHOA, PGF, SDC2, CCR2, MAPK14, IL1A, IL1R1, FOXO3, CP, ANXA5, ANGPT1, CXCR2, FLT1, CXCR4, NR3C1, C4A, CDC42, JAK2, EGR1, APOE, MMP14, TNFSF10, IGFBP1, CYBB, CRP, PCSK9, SELE, ESR1, F8, APOA2, NOS3, HRAS, INS, ACE, HSP90AA1, NLRP3, VEGFA, SERPINE1, APOA5, SOCS1, SELP, LCK, EGF, IGF1, ALB, ELN, ENG, ALDOA
3	10.897	79	425	NPC2, MMP12, PIK3R1, KEAP1, HSPA1A, PDGFA, CTSG, CDK6, ACAN, IMPDH1, MTTP, ARSA, SYP, CHEK1, PRL, SPARC, CHUK, GRN, CYP19A1, BMP4, IKBKG, CTSK, DNMT1, C3, TGM2, NR1H4, NAMPT, CYBA, BPI, GC, HEXB, CLU, LYZ, HP, SELPLG, TGFBR1, ANG, NPC1L1, ITGB2, FABP5, EPHA2, H2AFX, RNASE3, APOH, VTN, ABCG8, LMNA, RBP4, ABCG5, SYK, FGA, RNASE2, CYP7A1, VCL, BCAR1, LPA, SERPINA1, APOC3, POSTN, RAC1, VCP, GM2A, CSK, LIPG, SREBF2, APCS, BACE1, FGG, FGFR1, PLCG1, PTX3, SCARB1, ZAP70, RAC2, ITGB3, ADRB2, POMC, ABCG1, HSP90AB1
4	9.855	84	409	BGLAP, ABCB1, VWF, TTR, F2, APOC1, DCN, MET, HPRT1, ARG1, IRS1, JAK3, F5, GJA1, CTSB, ESR2, LCN2, RAF1, CD209, XIAP, HPGDS, AGTR2, CXCL16, APOA4, APLN, TXN, APOM, CCL11, AHSG, P2RY12, NFE2L2, NPY, PSAP, SOD2, IGFBP3, APOB, FGFR2, CETP, GFAP, KNG1, GNB3, PDGFB, LIPC, NQO1, NOX1, GGT1, LCAT, PTK2, ERBB4, IKBKB, PON1, SORL1, DUSP1, MSR1, NCF1, PIK3CA, JAG1, APOC2, VDR, PTPN1, PARP1, SOD1, SST, GSK3B, MMP8, MIF, THBD, CDK2, ITGAL, PLAT, CCNA2, CCL13, GSR, CASR, GPIHBP1, ALOX5, BTK, PLA2G7, ABL1, PLTP, MAPK10, AGER, AKT2, FGF1
5	6.704	55	181	ITGAV, GSTP1, PRKCA, CRH, HSPA8, AURKA, SQSTM1, GSTA3, LRP1, GCK, MAPT, GSTM1, GSTA1, CYP2C9, KAT2B, MAPK12, APAF1, CRYZ, CASP7, PLK1, ITGA2B, SREBF1, DUSP6, GSTM3, GSTM2, GSTM5, CYP3A5, ROCK1, HTR2A, LTA4H, MAPK7, CDA, P2RY1, CYP2C19, UCP2, CYP2C8, VIP, PRKACA, SAA1, S100A9, GCLM, PPARA, FABP4, CTSS, ALPL, GHRL, AKR1B1, MTHFR, HK1, CYP1A1, GCLC, GSS, SOD3, PSEN1, UTS2
6	5.2	26	65	TPI1, ADK, APRT, TXNRD2, PDE3B, PDE4D, GPI, EFEMP2, MFAP5, PNP, HAGH, CALM2, PRDX5, ACTC1, MTHFD1, UCK2, NME2, DCK, YARS, PSPH, PYGL, PDE4B, MYH11, SLC2A10, SORD, AHCY
7	4.8	6	12	EXO1, RECQL, PMS2, XRCC5, KIF11, FEN1
8	4.786	29	67	NR1H3, LDLR, COG2, APOA1, ABCA1, CANT1, CD14, LPL, COL3A1, ATIC, F7, OLR1, SPG7, ITGA2, ANGPTL3, F11, HMGCR, F10, TREM1, QPCT, CHIT1, LTF, DPP4, F2R, PPBP, HSPD1, ITIH4, SDC4, BGN
9	3.6	6	9	GFPT1, GALE, GFPT2, CLPP, HTRA1, UAP1
10	3.2	16	24	TGFBR2, RAB5A, GSTM4, G6PD, PDPK1, PIK3CB, ITK, INSR, CALM1, CTSD, ACTA2, GSTA4, GSTO1, SORT1, WAS, GLRX
11	3.143	15	22	DOT1L, HDAC8, ABCC1, RSAD2, TK1, DHFR, DHODH, GMPR2, SULT1E1, NT5M, TYMS, PPP1CC, FDPS, RNASEL, NR1I2
12	3	3	3	COX5A, TFPI, PROCR
13	3	15	21	MB, TNNI3, MYLK, NPPB, AGPAT2, FCAR, FKBP1A, PRKG1, BMPR2, GBA, EDNRA, NPC1, CALM3, BST1, ACE2
14	3	7	9	RPSA, TYMP, EPHX2, GART, WARS, CCT7, SHMT1
15	3	3	3	DDX39B, SNRPA, RAN
16	3	3	3	RFK, PPCDC, ENPP1
17	3	3	3	OAT, SRM, OTC
18	2.8	11	14	CYP4A11, MTR, GMPR, IMPDH2, UMPS, BHMT, MTRR, PLA2G10, CYP2J2, GLO1, PLA2G2A

Cluster 1 is mainly involved in inflammatory response and immune response. Cluster 2 is related to coagulation pathway, inflammatory reaction, NO metabolism, vascular remodelling and so on. Cluster 3 is mainly associated with lipid metabolism, platelet activation, inflammatory response, neutrophil chemotaxis, and proliferation of macrophages and smooth muscle cells. Cluster 4 is involved in the metabolism of HDL and LDL, inflammatory response and redox reaction. Cluster 5 is related to antioxidant effect of glutathione. Cluster 8 is associated with coagulation pathway, inflammatory reaction and foam cell formation. Cluster 10 is involved in antioxidant effect of glutathione. Cluster 12 is associated with coagulation pathways. Cluster 17 failed to return any human biological processes. Cluster 6, 7, 9, 11, 13, 14, 15, 16, 18 do not return AS‐related biological processes. (Table S5). The main biological processes in cluster 1 were used as an example shown in Figure 2B.

#### 3.3.3 Pathway of DSYM‐AS PPI network

After the pathway enrichment analysis, twenty‐seven AS‐related signalling pathways are obtained (Figure 2C). The P values, fold enrichment and count of these signalling pathways were shown in Figure 2D. The details are described in Table S6.

Current research shows that the process of AS mainly contains lipid infiltration.^77^ It contains the following three processes: (a) accumulation and modification of lipid particles under the arterial endothelium ^78^; (b) adhesion and migration of monocytes ^79^; (c) Mononuclear cells that migrate to the subendothelial cells subsequently differentiate into macrophages; modified LDL particles play an critical role in the formation of foam cells.^80^

Endothelial dysfunction is the primary and earliest link in the pathogenesis of AS.^81^ During the period of plaque proliferation and morphological changes of smooth muscle cells, the main event is the endothelial injury response; the lesions of AS during this period mainly involved changes in endothelial function, lipid deposition under the endothelium, and recruitment and aggregation of monocytes and lymphocytes.^80^ When atherosclerotic lesions progress to complex plaques, there is involvement of smooth muscle cells.^82^ The manifestation of vascular endothelial damage is that when vasodilation is impaired, the release of NO in endothelial cells and vascular wall is reduced, peroxide is increased, and oxygen ions are increased.^81^ When endothelial dysfunction is further aggravated, it may cause increased permeability; and low‐density lipoprotein and macrophages will release cytokines, which summon leucocytes to roll and adhere to endothelial cells, causing revascularization.^82^ Leucocytes (mainly monocytes) adhering to endothelial cells gradually penetrate into the wall through endothelial cells; then, monocytes become macrophages under the stimulation of cytokines, further forming foam cells, which promotes the release of cell growth factors, mediates lymphocyte aggregation at the site of injury, and lead to chronic inflammation.^83^, ^84^ This promotes smooth muscle proliferation and endothelial cell shedding, causing structural damage to endothelial cells.^83^, ^84^ In addition to monocytes, lymphocytes are also important in inflammatory cells. Helper T lymphocyte type 1 (Th1) secretes harmful Th1 cytokines, causing an inflammatory response and promoting the formation of atherosclerotic plaques.^85^ Antigen‐presenting cells also play a crucial role in the inflammatory response, which can regulate Th1 and regulatory T lymphocytes under the stimulation of oxLDL, heat shock proteins, bacteria and inflammatory reactions.^86^

The prediction results showed that DSYM is able to regulate several AS‐related biological processes and signalling pathways such as coagulation pathway, inflammatory reaction, NO metabolism, vascular remodelling, lipid metabolism, neutrophil chemotaxis, and proliferation of macrophages and smooth muscle cells, PI3K‐Akt signalling pathway, Complement and coagulation cascades, HIF‐1 signalling pathway, TNF signalling pathway, Neurotrophin signalling pathway, Adipocytokine signalling pathway, Insulin resistance, NF‐kappa B signalling pathway, Insulin signalling pathway, PPAR signalling pathway and so on. In order to validate the key targets, biological processes and signalling pathways in AS and DSYM‐AS biological networks, a series of animal experiments and proteomics experiments were conducted.

### 3.4 General observation

Before the experiment, there was no significant difference in the bodyweight between ApoE‐/‐ mice and the same strain C57BL/6L mice (*P* > .05), which was comparable. During the modelling process, the colour, activity, and eating and drinking of the mice in each group were normal and there was no difference. At the 12th week and the 20th week, the bodyweight of each group was significantly increased (*P* < .01), and the body mass growth of ApoE‐/‐ mice was more obvious than that of C57BL/6L mice (*P* < .01). At the end of the 20th week, the quality of the blank group and the model group still increased significantly (*P* < .01). Compared with the 12th weekend, the trend of body mass changes in the other experimental groups was not obvious. During the administration period after the model establishment, the mice in each group had less food intake than before, the hair colour was less lustrous, and the activity and drinking water were normal. In the low dose and high dose groups of DSYM, one mouse in each group died due to improper intragastric administration. Immediately after death, the thoracic and abdominal aorta was dissected, and pale yellow lipid streaks were observed, and then oxidized and melted soon. The number of mice that eventually entered the statistics was 12 in each group (see Table 4).

**Table 4:** Changes in body mass

Group	n	Before the experiment	At the end of the 12th week	At the end of the 20th week
Blank	12	20.6 ± 1.3	23.7 ± 1.5▼	25.9 ± 1.8▼▲
Model	12	20.1 ± 0.9	27.1 ± 1.7▼*	29.4 ± 0.7▼▲*
Simvastatin + aspirin	12	20.0 ± 1.2	26.6 ± 1.6▼*◇	27.4 ± 1.4▼△*◆
DSYM low dose	13/12	20.6 ± 1.5	27.2 ± 1.4▼*◇	27.9 ± 1.3▼△*◆
DSYM high dose	13/12	20.3 ± 1.5	27.3 ± 1.2▼*◇	27.6 ± 1.5▼△*◆

Note

(1) Within the group: compared with ‘before the experiment’ ▼ *P* < .01; compared with ‘at the end of the 12th week’, △ *P* > .05, ▲ *P* < .01. (2) Between groups: compared with the blank group * *P* ＜ .01; compared with the model group, ◇*P* ＞ .05, ◆ *P *＜ .01.

### 3.5 Changes in Serum NO, ET, Lipid, hs‐CRP, VEGF and MMP‐9 in ApOE‐/‐ Mice

#### 3.5.1 Effect of DSYM on serum NO and ET levels in ApOE‐/‐ mice

**Figure 3:**
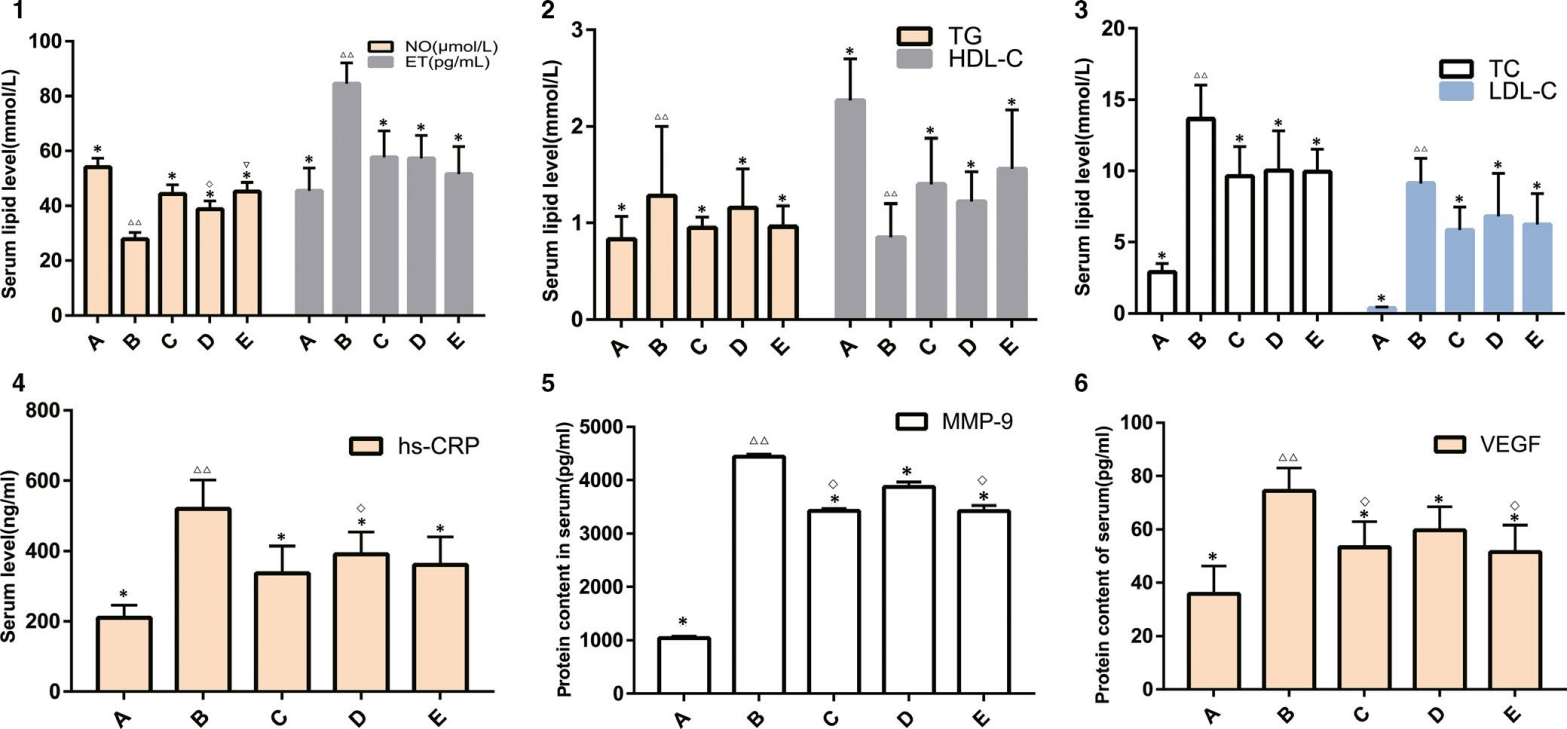
Results of Animal Experiments A, Effect of DSYM on Serum NO and ET Levels. B, Effect of DSYM on TC and HDL‐C. C, Effect of DSYM on TG and LDL‐C. D, Effect of DSYM on Serum hs‐CRP. E, Effect of DSYM on Serum VEGF. F, Effect of DSYM on Serum MMP‐9 (*compared with the model group, *P* < .05. △△ compared with the blank group, *P* < .01. ◇compared with the simvastatin + aspirin, *P* < .05. ▽compared with the DSYM low dose group, *P* < .05. A: blank group; B: model group; C: simvastatin + aspirin control group; D: DSYM low dose group; E: DSYM high dose group.)]

The serum NO level in the model group was significantly lower than that in the other group, and the ET content was significantly higher than that in the other group (*P* < .01). Drug intervention can improve NO and ET levels (*P* < .05), and the efficacy of DSYM was positively correlated with dose (*P* < .05). The DSYM high dose group was comparable to the simvastatin + aspirin control group in increasing NO (*P* > .05), but the DSYM group was superior to the western medicine group in reducing ET (*P* < .05). (Figure 3A).

#### 3.5.2 Effect of DSYM on serum lipid in ApOE‐/‐ mice

In the model group, the TC, TG, LDL‐C increased significantly, HDL‐C decreased significantly (*P* < .01), and the standard Western diet induced ApoE knockout mice to form severe hyperlipidemia. Compared with the model group, DSYM and simvastatin + aspirin can reduce TC and LDL‐C and increase HDL‐C (*P* < .05), but the lipid‐lowering effect of the three groups is not statistically significant (*P* > .05). This indicates that the DSYM group is equivalent to the conventional western medicine treatment group. The increases of DSYM dose had no significant effect on its curative effect of lipid‐lowering (*P* > .05). (Figure 3B, C).

#### 3.5.3 Effect of DSYM on serum hs‐CRP in ApOE‐/‐ mice

The hs‐CRP level in the model group was significantly higher than that in the blank group (*P* < .01), which was also higher than the other groups (*P* < .05). The hs‐CRP levels in the drug intervention group decreased, and that in the DSYM high dose group and the simvastatin + aspirin group decreased. There was no significant difference between DSYM high dose group and the simvastatin + aspirin group (*P* > .05) (Figure 3D).

#### 3.5.4 Effect of DSYM on serum VEGF and MMP‐9 in ApOE‐/‐ MIce

Serum VEGF and MMP‐9 levels in the model group were significantly higher than those in the group (*P* < .01); drug intervention could down‐regulate VEGF and MMP‐9 levels (*P* < .05 or *P* < .01). In the reduction of VEGF and MMP‐9, the efficacy of the DSYM high dose group was comparable to that of the simvastatin + aspirin group (*P* > .05), but better than the DSYM low dose group (*P* < .05). The efficacy of DSYM was dose dependent. (Figure 3E, F).

### 3.6 Effect of DSYM on pathomorphology of aortic root in ApOE‐/‐ mice

**Figure 4:**
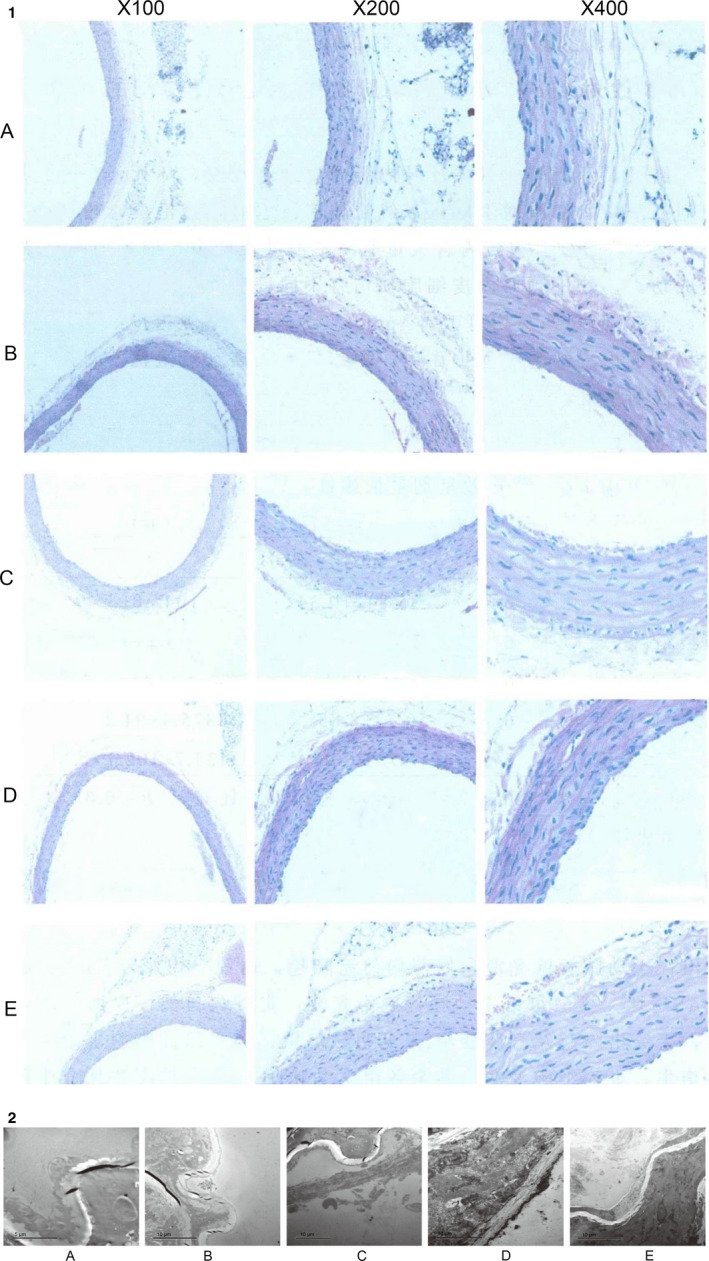
Pathological results Figure 4A: Pathomorphology of Aortic Root in each group. B, Ultrastructure of aortic vascular endothelial in each group (A: DSYM low dose group; B: Simvastatin + aspirin group; C: DSYM high dose group; D: model group; E: blank group)]

Blank group: the aortic wall was smooth, the thickness was uniform and no bulge, the endometrium, the media and the adventitia were not abnormal, and there was no AS lesion. Model group: the aortic wall is not smooth, the movement is not natural, the thickness is uneven, the intima is thickened, there is no protruding bulge and the lumen is narrowed, no obvious plaques are seen; Foam cell formation, smooth muscle cell proliferation, and inflammatory cell infiltration can be seen. The degree of change in the aorta of the remaining groups under HE staining was between that of the blank group and the model group (Figure 4A).

### 3.7 Effect of DSYM on ultrastructure of aortic vascular endothelial in ApOE‐/‐ mice

In the model group, the endometrial endothelial cells of the aorta were detached and necrotic, the structure of the internal elastic membrane was irregular; the smooth muscle cells proliferated obviously, the mitochondria were extensively oedematous, vacuolated, the sputum was significantly reduced or disappeared, the nuclear structure was blurred, and the cytoplasm contains a large number of lipid droplets or even a string, suggesting a lipid‐line stage, indicating successful modelling.

After drug intervention, endothelial cell injury was alleviated to varying degrees, indicating that DSYM, simvastatin + aspirin can improve the ultrastructure of aortic endothelial cells in ApoE‐/‐ mice, and protect the injured endothelial cells; The DSYM high dose group was superior to the control group, while the control group was superior to the DSYM low dose group. (Figure 4B).

### 3.8 QAM‐CAA‐4000 antibody protein chip data analysis

#### 3.8.1 Arrangement of 200 cytokines on membrane chips and expression profile of antibody chips

**Figure 5:**
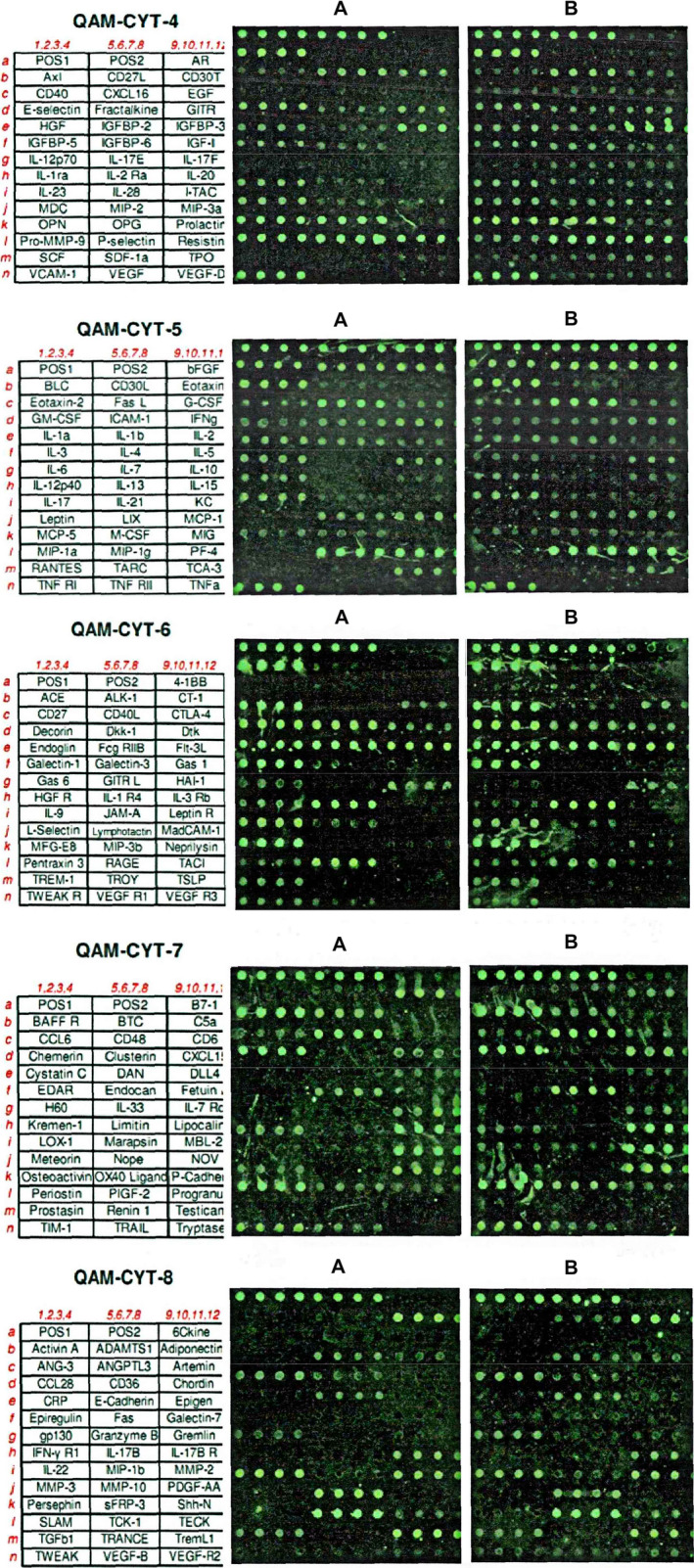
Arrangement and results of 200 cytokines on membrane chips

The arrangement and results of the 200 cytokines in the model group and the DSYM group on the membrane chip are shown in Figure 5.

#### 3.8.2 Antibody protein chip results data analysis

The results of the RayBio. Cytokine Antibody Arrays protein chip were analysed using the data of Normalization 2 Positive Control Normalization without Background. The signal value ratio method was used for chip analysis, and the difference between the signal value, Fold change and t test was selected. Select the signal value greater than 500, fold change greater than 2, less than 0.5 as the difference factor.

The analysis results of the two groups of sample chips showed that 51 models with significant differences were found in the model group/DSYM group, see Table S7. The 4‐1BB with the largest fold ratio has a low signal value in the model group, so it is rounded off. This study selected bFGF (whose signal value is maximum), Pro‐MMP‐9 (whose fold change ratio is maximum) and VEGF (which is closely related to vascular endothelial cell angiogenesis) for further study. (Table S7).

### 3.9 Expression of VEGF, MMP‐9 and bFGF mRNA in aortic lysates of each group

The expression of VEGF, MMP‐9 and bFGF mRNA in C57BL/6L mice was weak, and the expression levels of VEGF, MMP‐9 and bFGF mRNA in ApoE‐/‐ mice were enhanced; and the expression of model group was significantly higher than that of other groups (*P* < .01). The expression of MMP‐9 mRNA between DSYM low dose group and high dose group was statistically significant (*P* < .05). There was no significant difference in the expression of VEGF and bFGF mRNA among the drug intervention groups (*P* > .05; Figure S6).

To perform deep mining of proteomics data, we used systematic biological methods to further analyse proteomics data.

### 3.10 Experimental protein network analysis

#### 3.10.1 Experimental Protein Network

**Figure 6:**
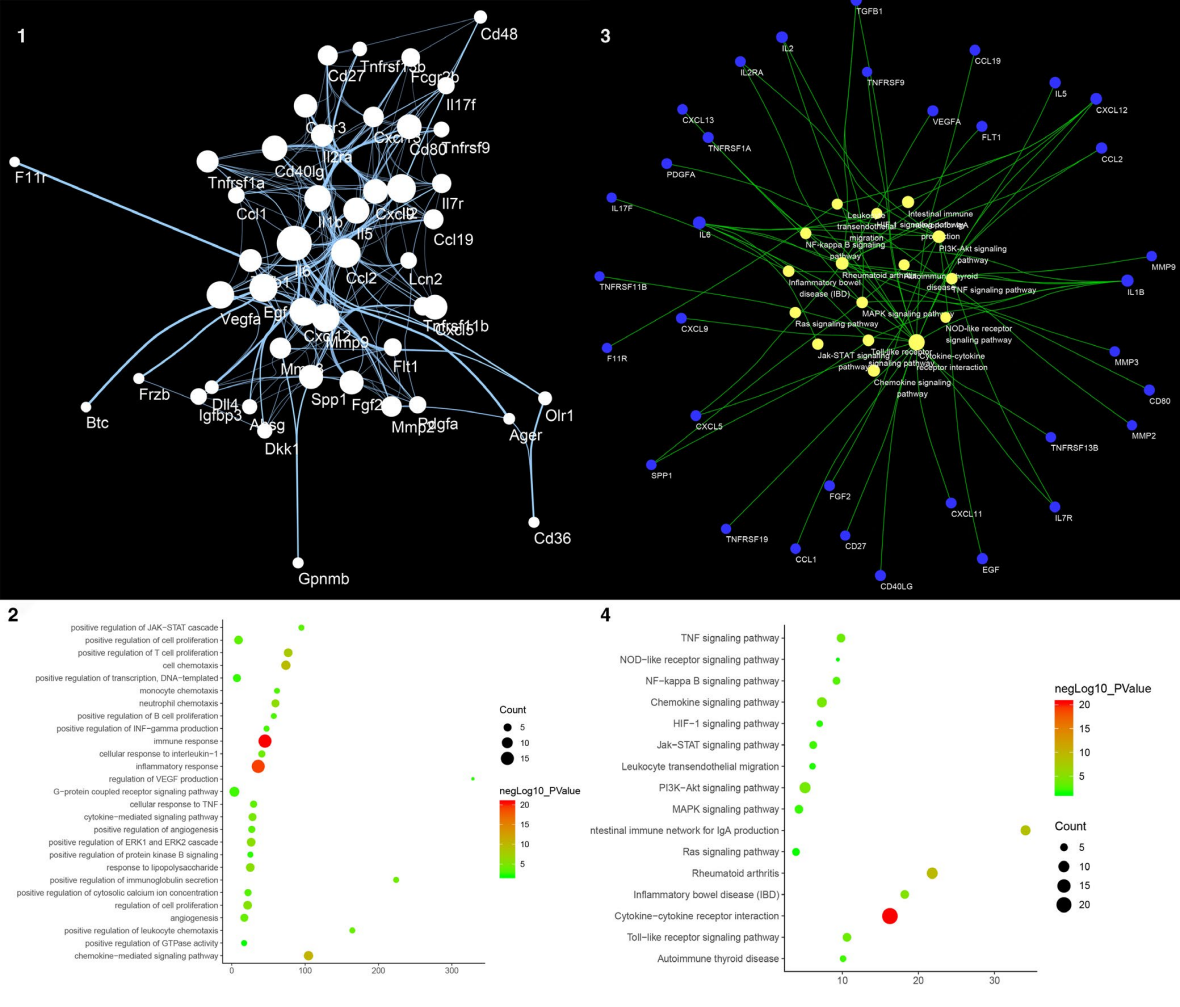
Results of Experimental Protein Network Analysis A, Experimental Protein Network (The larger the node size, the higher the degree of the node. The thicker the line, the greater the Edge Betweenness of the node.). B, Bubble chart of biological processes (X‐axis is fold enrichment analysis). C, Pathway of Experimental Protein Network (Blue circle stands for proteins. Yellow circle stands for pathway. The larger the node size, the higher the degree of the node. The thicker the line, the greater the Edge Betweenness of the node.). D, Bubble chart of signalling pathway (X‐axis is fold enrichment analysis)

The experimental proteins and their PPI network were utilized to construct the experimental protein network. This network contains 46 nodes and 350 edges (Figure 6A).

#### 3.10.2 Biological Processes of Experimental Protein Network

The experimental protein network was analysed by MCODE and returns 2 clusters (Table 5 and Figure S7). The genes in the clusters were input into DAVID for GO enrichment analysis.

**Table 5:** Cluster of experimental protein network

Cluster	Score	Nodes	Edges	Proteins
1	14.857	22	156	Cxcr3, Il5, Tnfrsf11b, Cxcl9, Tnfrsf1a, Il6, Fcgr2b, Egf, Il1b, Cxcl13, Cxcl12, Spp1, Ccl1, Ccl2, Cxcl5, Mmp2, Fgf2, Mmp3, Cd40lg, Cd80, Ccl19, Il2
2	3.6	11	18	Il17f, Flt1, Tnfrsf9, Mmp9, Igfbp3, Tgfb1, Il7r, Cd27, Il2ra, Vegfa, Lcn2

Cluster 1 is mainly involved in immune response (such as regulatory T cells, neutrophil chemotaxis), inflammatory response, angiogenesis and vascular endothelial barrier. Cluster 2 is associated with inflammatory responses, angiogenesis, cellular hypoxia and immune responses (Table S8). The main biological processes in cluster 1 were used as an example shown in Figure 6B.

#### 3.10.3 Pathways of Experimental Protein Network

After the pathway enrichment analysis, sixteen AS‐related signalling pathways are obtained (Figure 6C). The P values, fold enrichment and count of these signalling pathways were shown in Figure 6D. The details are described in Table S9.

### 3.11 Experimental protein‐other proteins’ ppi network analysis

#### 3.11.1 Experimental protein‐other proteins’ PPI network

**Figure 7:**
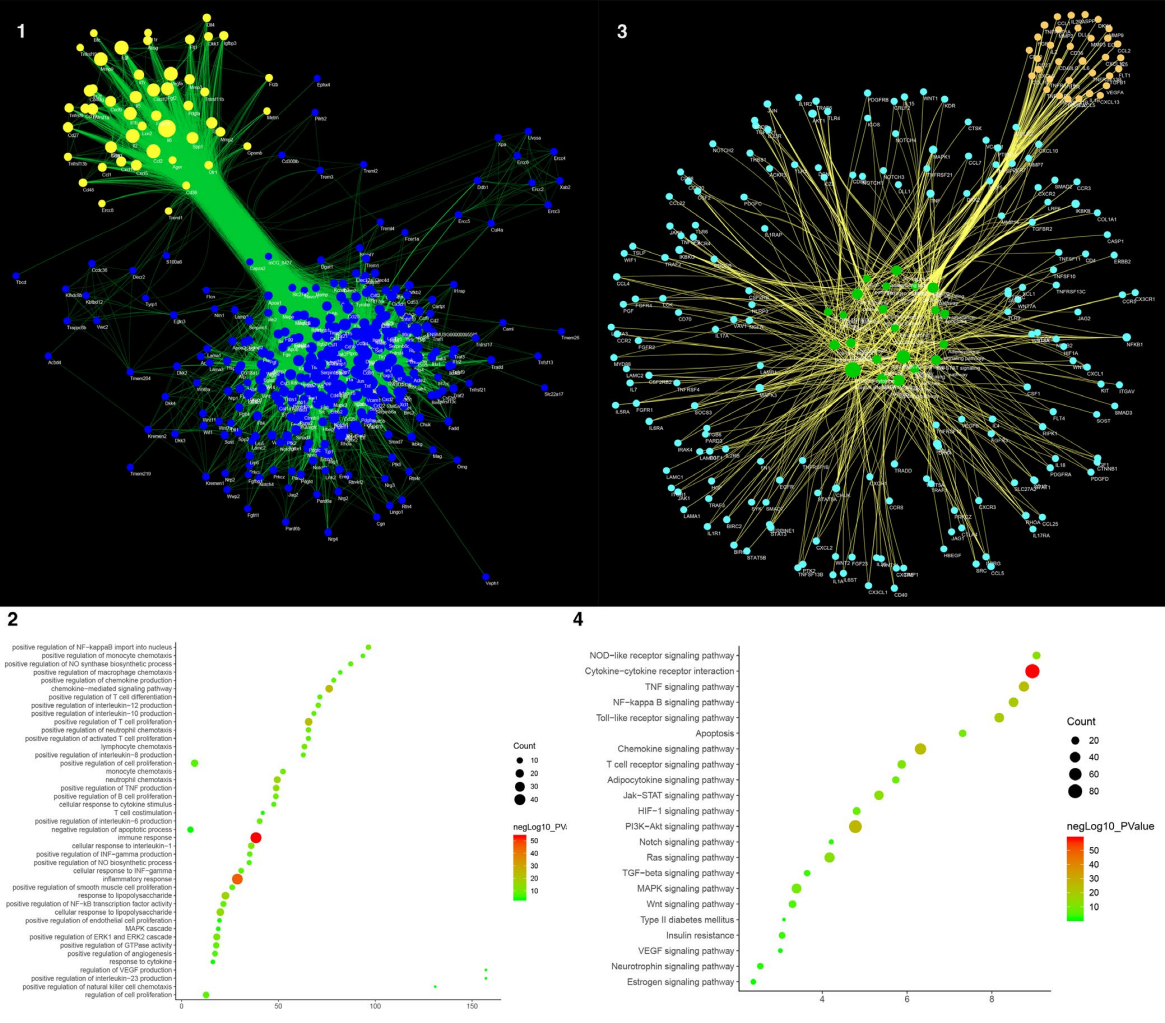
Results of Experimental Protein‐Other Proteins’ PPI Network Analysis, A, Experimental Protein‐Other Proteins’ PPI Network (Green circle stands for experimental protein. Blue circle stands for other protein. The larger the node size, the higher the degree of the node. The thicker the line, the greater the Edge Betweenness of the node.). B, Bubble chart of biological processes (X‐axis is fold enrichment analysis). C, Pathway of Experimental Protein‐Other Proteins’ PPI Network (Blue circle stands for other protein. Yellow circle stands for experiment protein. Green circle stands for pathway. The larger the node size, the higher the degree of the node. The thicker the line, the greater the Edge Betweenness of the node.). D, Bubble chart of signalling pathways (X‐axis is fold enrichment analysis)

The other proteins of experimental protein were obtained from String. The other proteins, experimental proteins and their PPI network were utilized to construct the experimental protein‐other proteins’ PPI network. This network contains 374 nodes and 10 392 edges (Figure 7A).

#### 3.11.2 Biological processes of experimental protein‐other proteins’ PPI network

The experimental protein‐other proteins’ PPI Network was analysed by MCODE and returns 13 clusters (Table 6; Figure S8). The genes in the clusters were input into DAVID for GO enrichment analysis.

**Table 6:** Cluster of experimental protein‐other proteins’ PPI network

Cluster	Score	Nodes	Edges	Proteins
1	62.423	72	2216	Cxcl5, Cxcl1, Itgam, Cd86, Sell, Cd44, Il1b, Ccr3, Tbx21, Il7, Il7r, Cxcl2, Il15, Ccl7, Ccl22, Fcgr2b, Alb, Cd28, Stat1, Cx3cr1, Cx3cl1, Ccl20, Vcam1, Il4, Csf1, Cd19, Ccr5, Il18, Ccl5, Serpinb6e, Foxp3, Serpinb6b, Serpinb6a, Serpinb6c, Cxcr3, Tlr4, Cxcl10, Tlr2, Myd88, Ccr2, Ccl2, Cd40lg, Cxcr5, Cd40, Cxcl13, Tlr9, ENSMUSG00000095585, Ccr7, Cxcl9, Akt1, Il17a, Ccl4, Il5, Nlrp3, Cd80, Cd274, Ctla4, Tnf, Ptprc, Il2, Il2ra, Stat5a, Cd27, Cxcl12, Cxcr4, Tnfrsf1a, Itgax, Stat3, Csf2, Il6, Cxcr2, Ccl19
2	19.831	72	704	Tnfsf9, Notch1, Fpr2, Apob, Dmp1, Il1a, Mmp12, Itih2, Wnt2, Afp, Adam10, Jak1, Mapk1, Lamc1, Hgf, Il23r, Ahsg, Mmp8, Hif1a, Igfbp3, Mmp14, Pdgfrb, Pdgfb, Figf, Jak3, Runx2, Spp2, Il22, Serpinc1, Rhoa, Serpine1, Tyrobp, Vegfc, Fgf23, Ackr3, Smad2, Fga, Fgg, Ptk2, Il1r1, Flt1, Pgf, Src, Rorc, Egf, Egfr, Jag1, Mmp2, Mepe, Ctnnb1, Wnt5a, Il17ra, Lgals3, Nfkb1, Kdr, Tgfb1, Mmp7, Smad3, Sdc2, Apoa1, Mmp10, Apoa2, Igfbp7, Tnfrsf1b, Cst3, Bglap, Fgf2, Itgb1, Lamb1, Igf2, Vegfb, Cd70
3	11.65	41	233	Il6ra, Tnfrsf9, Casp1, Flt3, Rela, Spp1, Icos, Cybb, Tnfsf11, Syk, Tlr6, Plg, Fn1, Jun, Kit, Itgal, Mapk3, Ccl25, Xcl1, Cxcr1, Alpi, Il2rb, Tnfrsf4, Il2rg, Il17f, Cd38, Tnfsf13b, Traf6, Ccl1, Socs3, Vegfa, Ccr8, Hmgb1, Lck, Tslp, Cd4, Igf1, Mmp3, Mmp9, Jak2, Timp1
4	11	11	55	Ercc8, Ercc2, Xpa, Ercc3, Ercc4, Ddb1, Ercc6, Cul4a, Ercc5, Uvssa, Xab2
5	8.75	9	35	Metrn, Vwc2, Klhdc8b, Tmem204, Egln3, Kbtbd12, Trappc6b, Ccdc36, Decr2
6	7.243	38	134	Erbb2, Traf2, Cd244, Flt4, Tnfrsf11b, Tnfsf10, Dcn, Vav1, Traf3, Hbegf, Erbb3, Lcn2, Fgf1, Itgb2, Birc3, Birc2, App, Ripk1, Tradd, Wnt1, Chuk, Pdgfa, Timp2, Ikbkg, Stat5b, Ikbkb, Ptpn11, Col18a1, Nfkb2, Il5ra, Thbs1, Dkk1, Wnt3a, Timp3, Col1a1, Pdgfra, Ltf, Bglap2
7	6.571	8	23	Frzb, Wnt11, Dkk3, Wnt8a, Wnt7a, Wif1, Sost, Dkk2
8	6	6	15	Olr1, Kcnab2, Itgav, Cd36, Slc27a2, Dgat1
9	6	6	15	Rtn4r, Tnfrsf19, Rtn4, Mag, Lingo1, Omg
10	4.75	9	19	Ereg, Nrg3, Pdgfd, Nrg2, Fgfr3, Nrg4, Btc, Ptk6, Fgfr4
11	4	5	8	Pard6a, Prkci, Pard6b, Cgn, F11r
12	3.167	13	19	Notch2, Nrp1, Erbb4, Nrg1, Diap1, Fgfr1, Pth, Dll4, Notch3, Ctsk, Ngfr, Pdgfc, Lrp5
13	3	3	3	Treml1, Trem3, Pilrb2

Cluster 1 is associated with immune reactions, inflammatory responses, angiogenesis, neutrophil chemotaxis, proliferation of monocyte macrophages and smooth muscle. Cluster 2 is associated with Proliferation and migration of smooth muscle, angiogenesis, and proliferation of endothelial cells and their signalling pathways. Cluster 3 is mainly related to inflammatory responses. Cluster 6 is also involved in the inflammatory response. Cluster 7 is associated with Wnt signalling pathway. Cluster 8 is primarily involved in lipid metabolism. Cluster 5 and 13 do not return any human's biological processes. Cluster 4, 9, 10, 11, 12 failed to return AS‐related biological processes (see Table S10). The main biological processes in cluster 1 were used as an example shown in Figure 7B.

#### 3.11.3 Pathway of experimental protein‐other proteins’ PPI network

After the pathway enrichment analysis, twenty‐two AS‐related signalling pathways are obtained (Figure 7C). The P values, fold enrichment and count of these signalling pathways were shown in Figure 7D. The details are described in Table S11.

Comparing the pathways of experimental protein network and experimental protein‐Other proteins’ PPI network, it is found that the AS‐related pathways they share are Cytokine‐cytokine receptor interaction, PI3K‐Akt signalling pathway, Chemokine signalling pathway, TNF signalling pathway, NF‐kappa B signalling pathway, Toll‐like receptor signalling pathway, Jak‐STAT signalling pathway, NOD‐like receptor signalling pathway, Ras signalling pathway, MAPK signalling pathway and HIF‐1 signalling pathway. The AS‐related pathways that experimental protein‐other proteins’ PPI network contains only are as follows: T‐cell receptor signalling pathway, Apoptosis, Adipocytokine signalling pathway, Wnt signalling pathway, TGF‐beta signalling pathway, Insulin resistance, Notch signalling pathway, Neurotrophin signalling pathway, VEGF signalling pathway, Oestrogen signalling pathway and Type II diabetes mellitus.

In addition, comparing the pathways of DSYM‐AS PPI network and experimental protein network, it is found that the AS‐related pathways they share are PI3K‐Akt signalling pathway, HIF‐1 signalling pathway, TNF signalling pathway, NF‐kappa B signalling pathway, Toll‐like receptor signalling pathway, MAPK signalling pathway, Leucocyte transendothelial migration, NOD‐like receptor signalling pathway, cytokine‐cytokine receptor interaction and Jak‐STAT signalling pathway. The AS‐related pathways that DSYM‐AS PPI network contains only are as follows: complement and coagulation cascades, Neurotrophin signalling pathway, Adipocytokine signalling pathway, Insulin resistance, Insulin signalling pathway, PPAR signalling pathway, VEGF signalling pathway, Type II diabetes mellitus, Oestrogen signalling pathway, Platelet activation, Apoptosis, mTOR signalling pathway, AMPK signalling pathway, Glutathione metabolism, Arachidonic acid metabolism, TGF‐beta signalling pathway and Glucagon signalling pathway.

The process of erosion or rupture of AS plaques involves a variety of inflammatory mechanisms including endothelial dysfunction, leucocyte migration, extracellular matrix degradation and platelet activation.^87^ The main mechanism is: (1) Cytokines that regulate leucocyte activity in acute phase reactants, such as interleukin (IL)‐6, 10, 18, monocyte chemoattractant protein (MCP)‐1, tumour necrosis factor (TNF)‐α, C‐reactive protein (CRP), serum amyloid A (SAA), may lead to the formation of AS plaques.^88^, ^89^ (2) Products of the acute phase of inflammation, such as soluble intercellular adhesion molecules (sICAM), soluble vascular cell adhesion molecules (sVCAM) and soluble E‐selectin (sE‐selectin, E‐S) in adhesion molecules can promote monocyte adhesion and leucocyte infiltration into the extravascular space, suggesting the degree of activation of endothelial cells.^90^, ^91^ (3) Plasma levels of endothelial cell activation and leucocyte adhesion markers such as myeloperoxidase (MPO), secreted phospholipase A2 (sPLA2) and lipoprotein‐associated phospholipase A2 (Lp‐PLA2) may reflect the degree of oxidative stress in AS plaques.^92^ (4) Oxidative stress markers such as vascular endothelial growth factor (VEGF), placental growth factor (PLGF) and hepatocyte growth factor (HGF) are potent vascular growth factors that are prone to plaque instability.^93^ (5) Inflammation in the plaque also results in the activation of metalloproteinases (MMPs), which decompose the extracellular matrix components of the fibrous caps such as collagen and elastic fibres, degrading the fibrous caps, and thus stabilizing the plaques.^94^, ^95^ (6) Platelet activation and aggregation markers such as sCD40L, soluble P‐selectin (P‐S) levels, and unstable plaque rupture and/or erosion can lead to thrombosis. Platelets are also activated before significant ACS appears, which directly leads to the development of AS.^96^, ^97^, ^98^ Our research showed that DSYM can interfere with these inflammatory molecules and their mediated biological processes.

## 4 CONCLUSION

This study proposed a new method based on systems biology, proteomics, and experimental pharmacology, and analysed the pharmacological mechanism of DSYM. DSYM may achieve therapeutic effects by regulating AS‐related biological processes (such as coagulation pathway, inflammatory reaction, NO metabolism, vascular remodelling, lipid metabolism, neutrophil chemotaxis, and proliferation of macrophages and smooth muscle cells) and signalling pathways (such as PI3K‐Akt, HIF‐1, TNF, neurotrophin, adipocytokine signalling pathways, and complement and coagulation cascades).

## CONFLICT OF INTEREST

We declare no competing interests.

## AUTHOR CONTRIBUTION

**Kailin Yang:** Conceptualization (lead); Data curation (equal); Formal analysis (equal); Methodology (equal); Software (lead); Visualization (lead); Writing‐original draft (equal). **Liuting Zeng:** Conceptualization (lead); Data curation (equal); Formal analysis (equal); Methodology (equal); Software (lead); Visualization (supporting); Writing‐original draft (equal). **Anqi Ge:** Conceptualization (supporting); Data curation (equal); Formal analysis (equal); Methodology (equal); Writing‐original draft (equal). **Xiaoping Pan:** Conceptualization (supporting); Data curation (equal); Formal analysis (equal); Methodology (equal); Writing‐original draft (equal). **Tingting Bao:** Conceptualization (supporting); Data curation (equal); Formal analysis (equal); Methodology (equal); Writing‐original draft (equal). **Zhiyong Long:** Conceptualization (supporting); Data curation (equal); Formal analysis (equal); Methodology (equal); Writing‐original draft (equal). **Qiaozhen Tong:** Data curation (equal); Formal analysis (equal); Methodology (equal). **Mengxia Yuan:** Data curation (supporting); Formal analysis (supporting); Methodology (supporting). **Xiaofei Zhu:** Data curation (supporting); Formal analysis (supporting); Methodology (supporting). **Jinwen Ge:** Conceptualization (supporting); Data curation (equal); Formal analysis (equal); Funding acquisition (equal); Methodology (equal); Supervision (equal); Validation (equal); Writing‐review & editing (equal). **Zhengde Huang:** Conceptualization (supporting); Data curation (equal); Formal analysis (equal); Funding acquisition (equal); Methodology (equal); Supervision (equal); Validation (equal); Writing‐review & editing (equal).

## Supporting information

Figure S1

Figure S2

Figure S3

Figure S4

Figure S5

Figure S6

Figure S7

Figure S8

Table S1

Table S2

Table S3

Table S4

Table S5

Table S6

Table S7

Table S8

Table S9

Table S10

Table S11

Fig S1Legend

Fig S2Legend

Fig S3Legend

Fig S4Legend

Fig S5Legend

Fig S6Legend

Fig S7Legend

Fig S8Legend

## Data Availability

The data that support the findings of this study are openly available in supplementary materials.
