# Supplementary figures and images for: Integrating systematic biological and proteomics strategies to explore the pharmacological mechanism of danshen yin modified on atherosclerosis
Source: J Cell Mol Med. 2020 Nov 2;24(23):13876–98. doi: 10.1111/jcmm.15979 (PMC7753997; doi:10.1111/jcmm.15979)

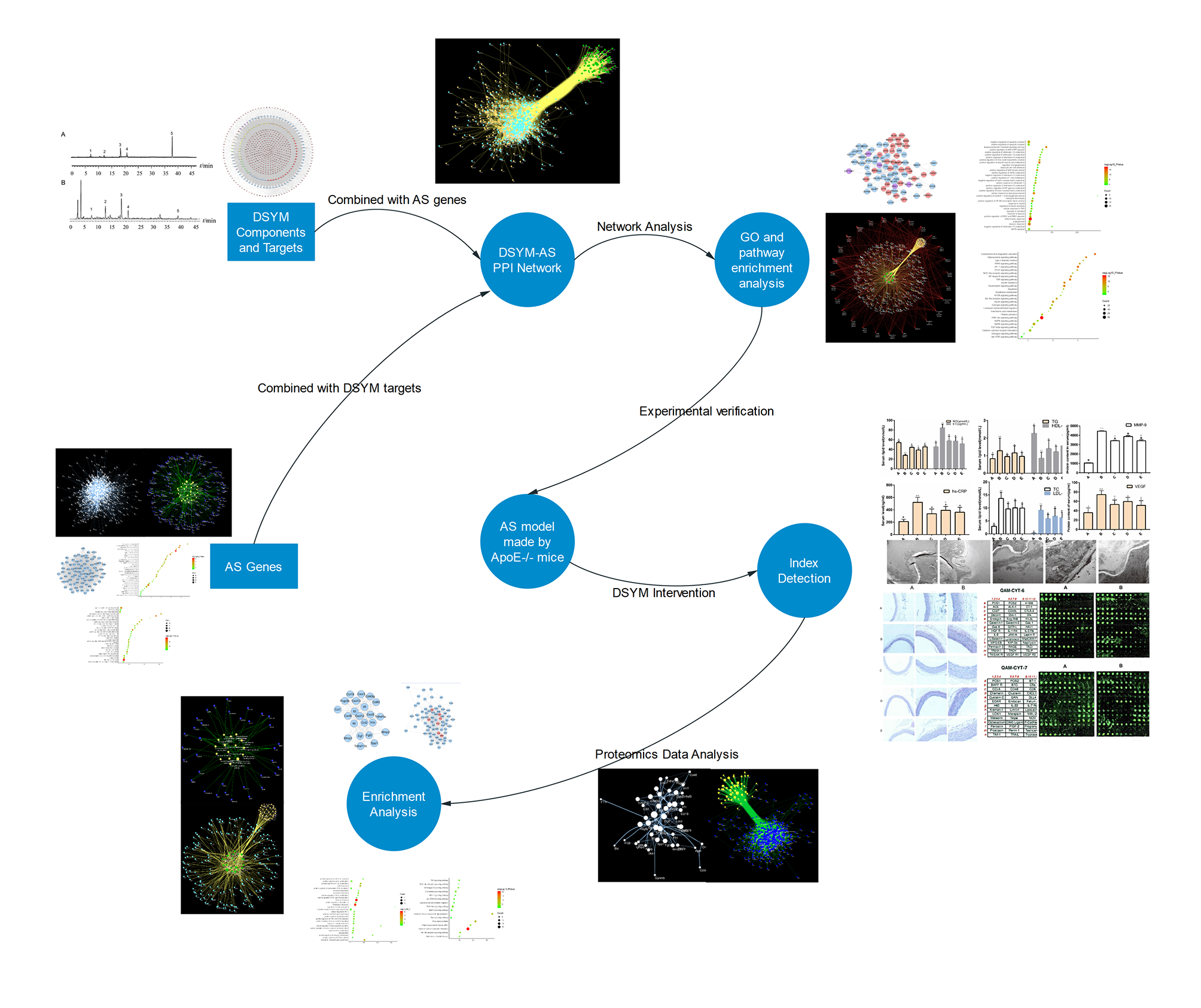

Supplement: Supplementary file 1 — Figure S1 [file JCMM-24-13876-s001.tif]

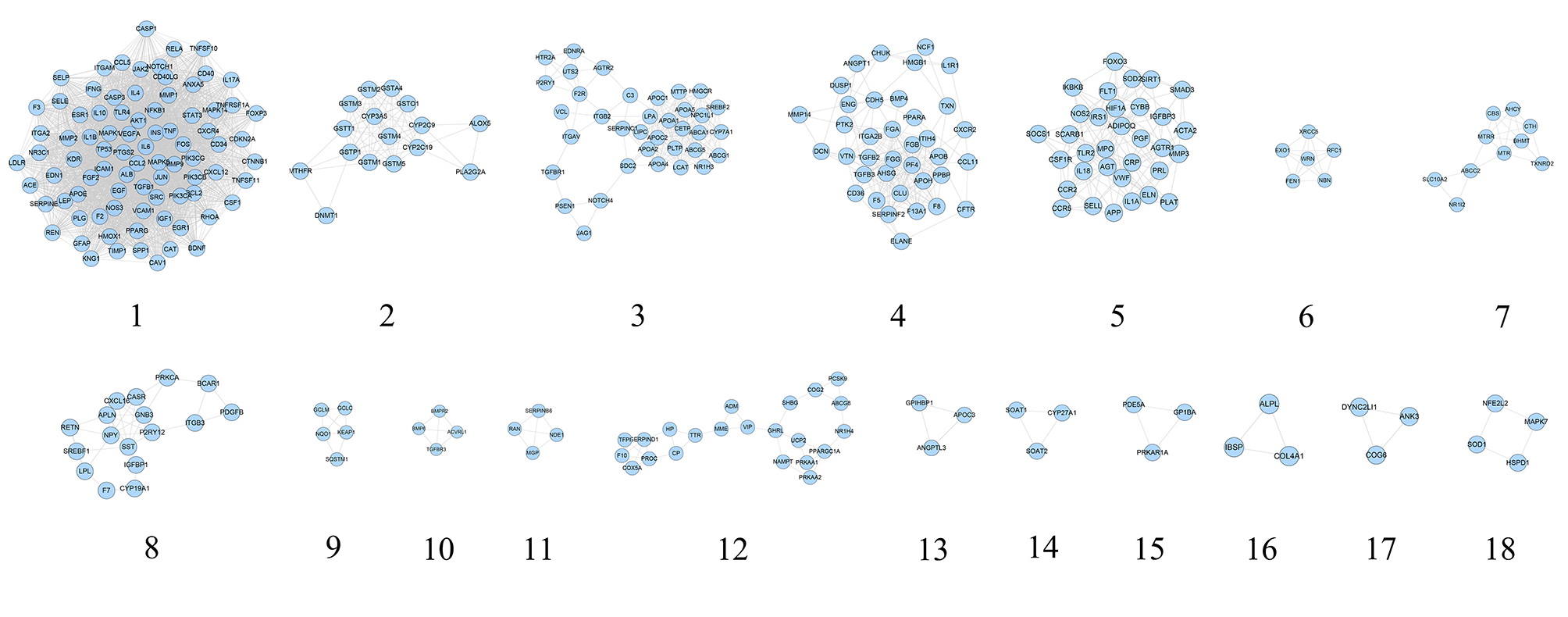

Supplement: Supplementary file 2 — Figure S2 [file JCMM-24-13876-s002.tif]

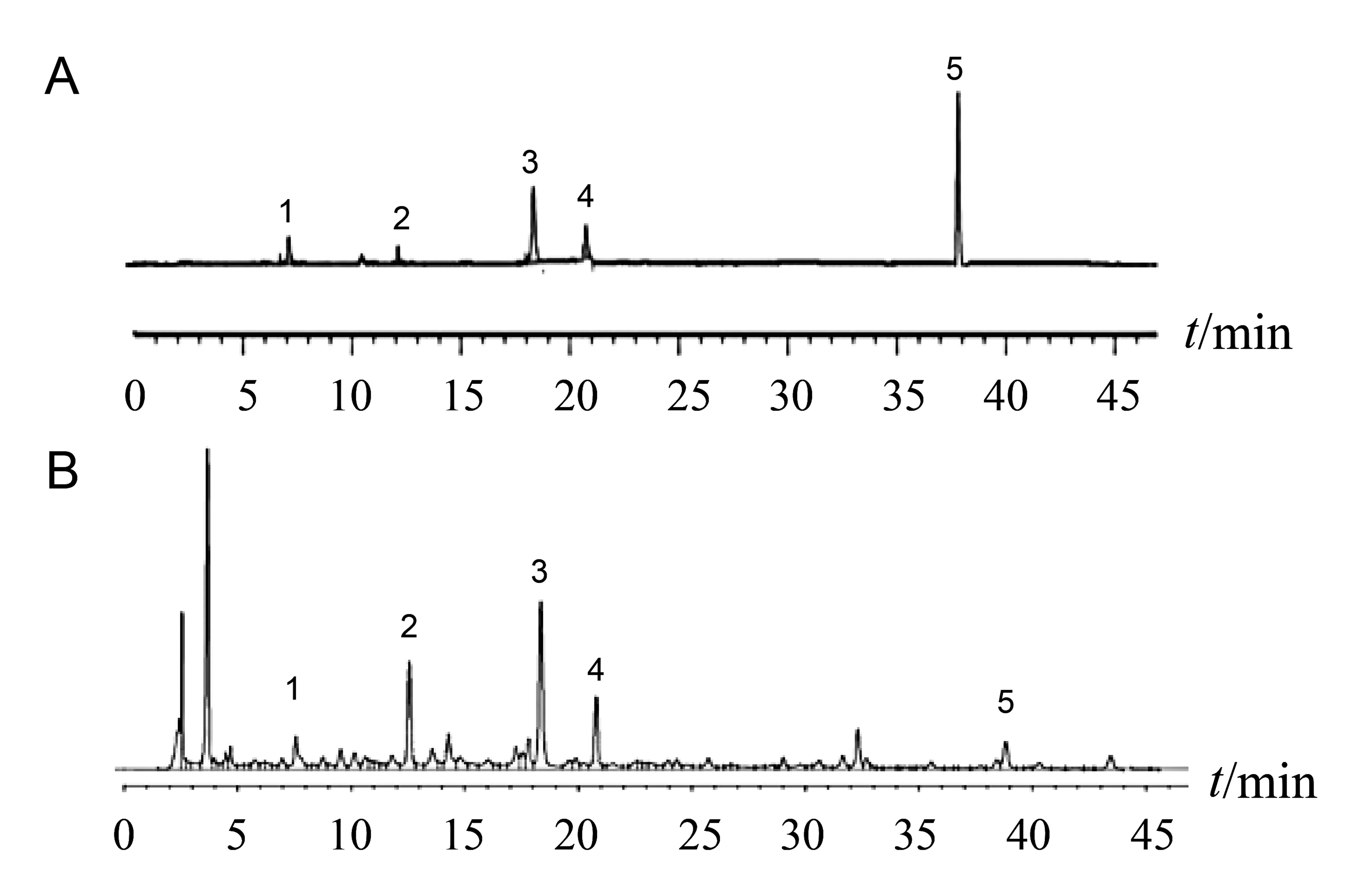

Supplement: Supplementary file 3 — Figure S3 [file JCMM-24-13876-s003.tif]

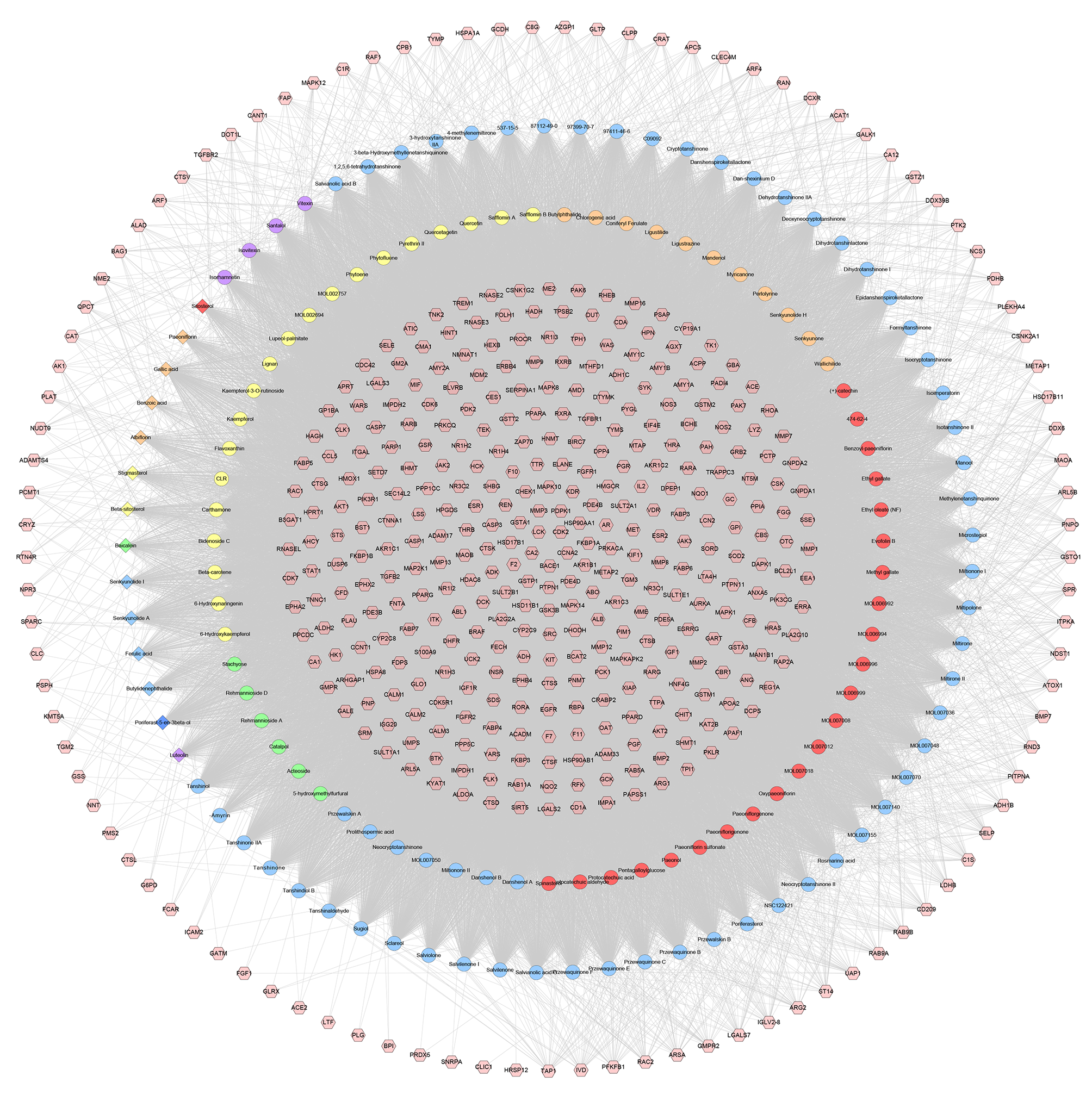

Supplement: Supplementary file 4 — Figure S4 [file JCMM-24-13876-s004.tif]

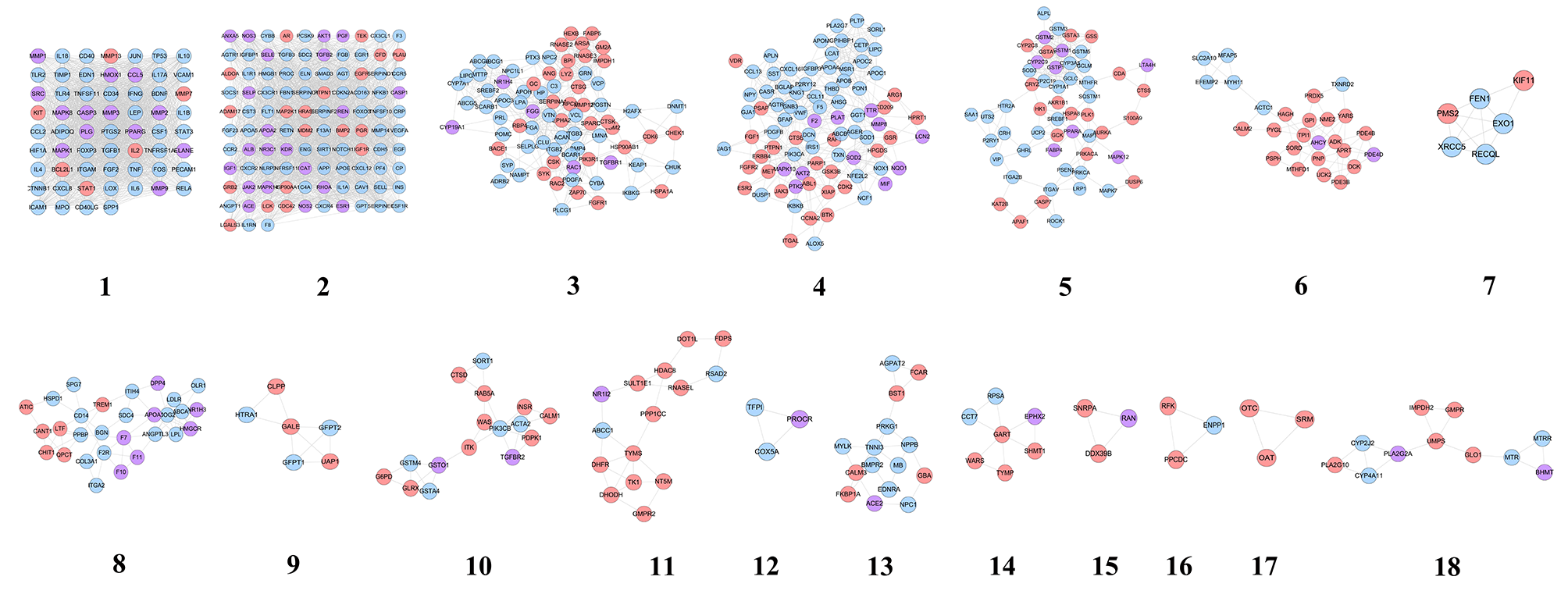

Supplement: Supplementary file 5 — Figure S5 [file JCMM-24-13876-s005.tif]

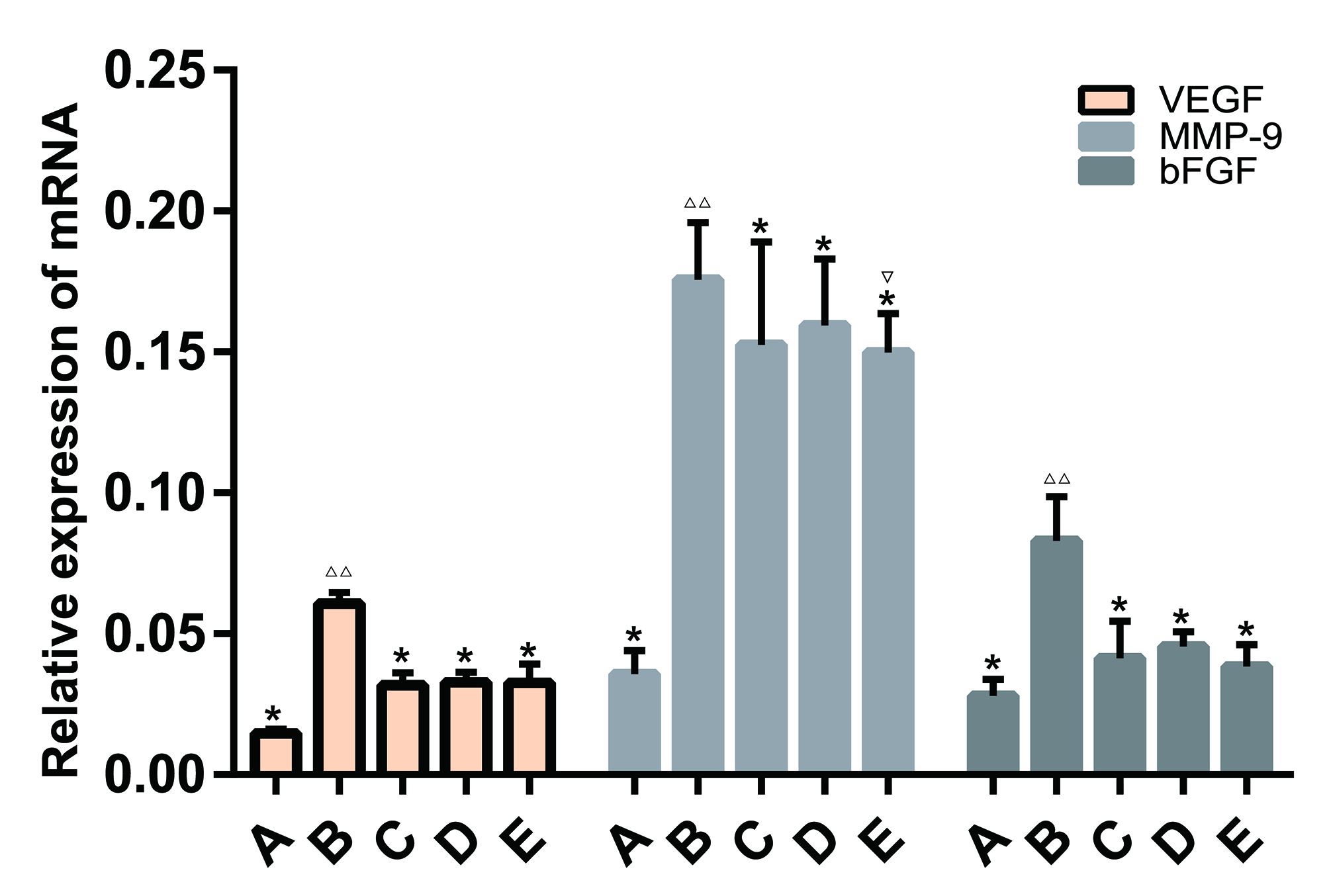

Supplement: Supplementary file 6 — Figure S6 [file JCMM-24-13876-s006.tif]

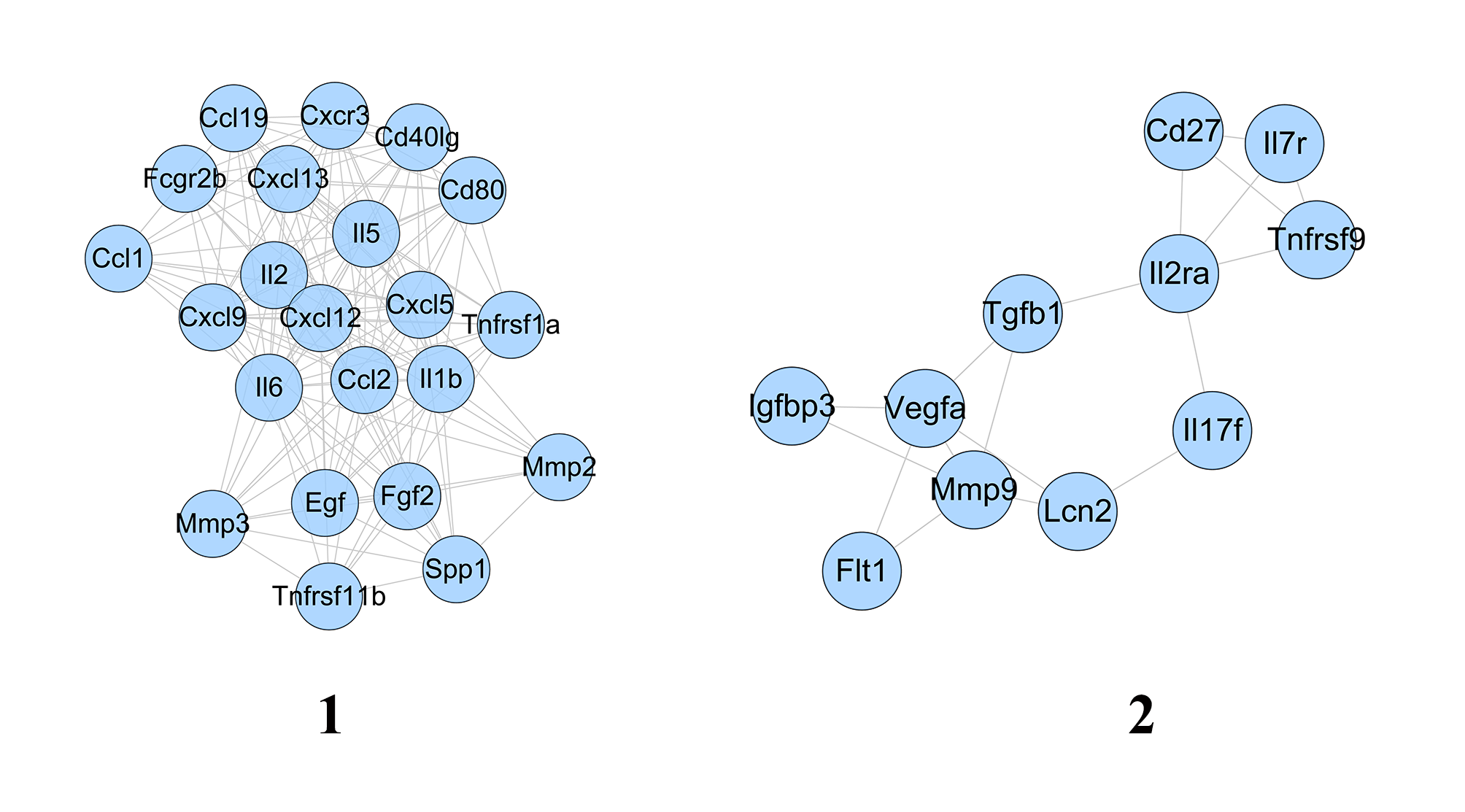

Supplement: Supplementary file 7 — Figure S7 [file JCMM-24-13876-s007.tif]

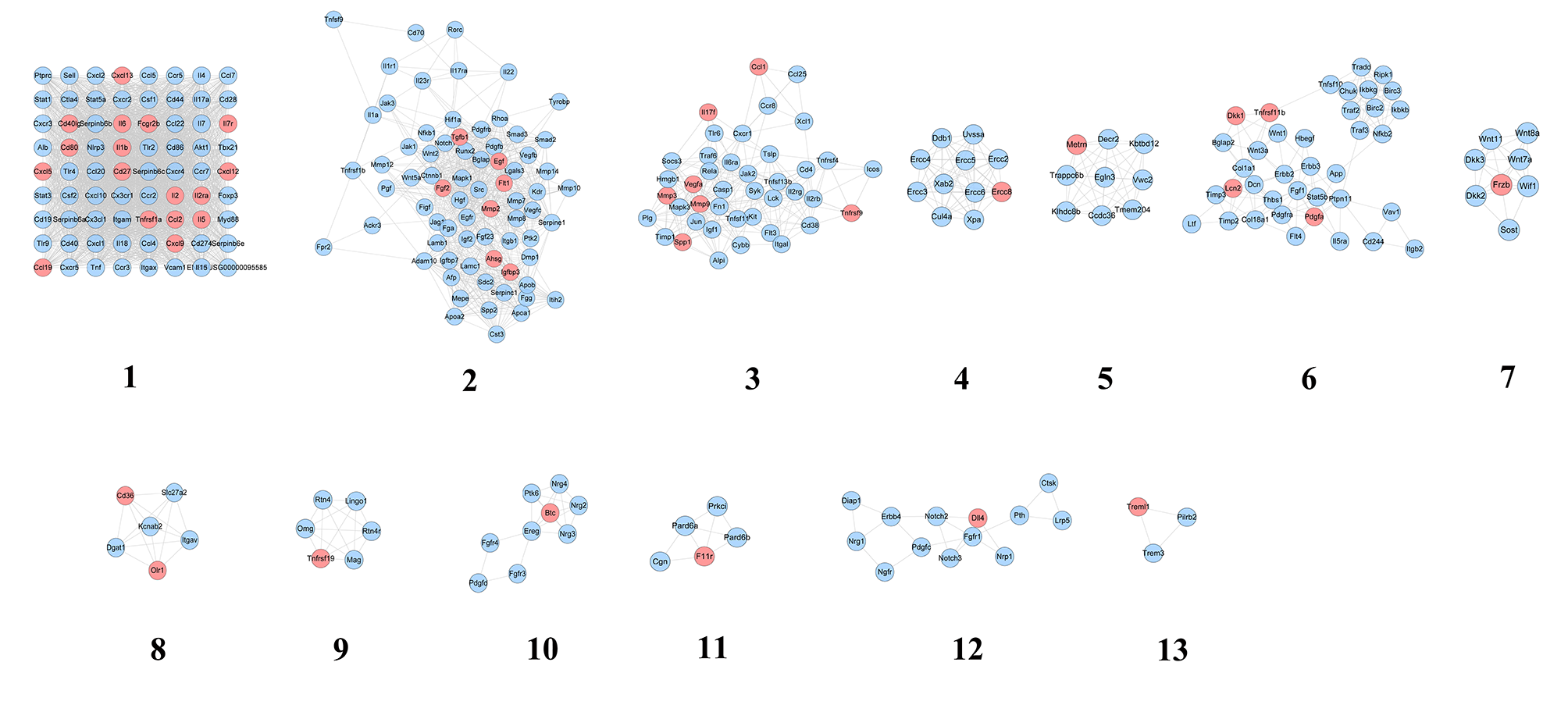

Supplement: Supplementary file 8 — Figure S8 [file JCMM-24-13876-s008.tif]
