# Supplementary material for: Integrating systematic biological and proteomics strategies to explore the pharmacological mechanism of danshen yin modified on atherosclerosis
Source: J Cell Mol Med. 2020 Nov 2;24(23):13876–98. doi: 10.1111/jcmm.15979 (PMC7753997; doi:10.1111/jcmm.15979)
Supplement: Supplementary file 22 — Fig S3Legend [file JCMM-24-13876-s022.docx]

Figure S3 DSYM Fingerprint (A: Standard sample; B: DSYM sample. 1: tanshinol; 2: protocatechuic aldehyde; 3: salvianolic acid B; 4: rosmarinci acid; 5: tanshinone IIA.)
