# Supplementary material for: Integrating systematic biological and proteomics strategies to explore the pharmacological mechanism of danshen yin modified on atherosclerosis
Source: J Cell Mol Med. 2020 Nov 2;24(23):13876–98. doi: 10.1111/jcmm.15979 (PMC7753997; doi:10.1111/jcmm.15979)
Supplement: Supplementary file 23 — Fig S4Legend [file JCMM-24-13876-s023.docx]

Figure S4 Compound-compound target network of DSYM consist of 581 compound target nodes and 140 compound nodes (Pink hexagon stand for compound targets; red, orange, yellow, green, blue and purple circles stand for compounds of *Radix Paeoniae Rubra*, *Chuanxiong Rhizoma*, *Carthami Flos*, *Rehmanniae Radix Praeparata*, *Radix Salviae*, *Santalum Album L.*, resp. Red diamond stands for common compound of *Radix Paeoniae Rubra* and *Chuanxiong Rhizoma*. Orange diamond stands for common compound of *Radix Paeoniae Rubra and Angelicae Sinensis Radix*. Yellow diamond stands for common compound of *Radix Paeoniae Rubra*, *Angelicae Sinensis Radix, Rehmanniae Radix Praeparata and Carthami Flos.* Green diamond stands for common compound of *Radix Paeoniae Rubra* and *Carthami Flos.* Blue diamond stands for common compound of *Chuanxiong Rhizoma* and *Angelicae Sinensis Radix.* Indigo-blue diamond stands for common compound of *Radix Salviae* and *Carthami Flos.* Purple diamond stands for common compound of *Santalum Album L., Radix Salviae* and *Carthami Flos.*)
