# Supplementary material for: Integrating systematic biological and proteomics strategies to explore the pharmacological mechanism of danshen yin modified on atherosclerosis
Source: J Cell Mol Med. 2020 Nov 2;24(23):13876–98. doi: 10.1111/jcmm.15979 (PMC7753997; doi:10.1111/jcmm.15979)
Supplement: Supplementary file 25 — Fig S6Legend [file JCMM-24-13876-s025.docx]

Figure S6 Expression of VEGF, MMP-9 and bFGF mRNA in aortic lysates of each group (*compared with the model group, P < 0.05. △△ compared with the blank group, P < 0.01. ▽compared with the DSYM low dose group, P < 0.05. A: blank group; B: model group; C: simvastatin + aspirin control group; D: DSYM low dose group; E: DSYM high dose group.)
