# Supplementary material for: Integrating systematic biological and proteomics strategies to explore the pharmacological mechanism of danshen yin modified on atherosclerosis
Source: J Cell Mol Med. 2020 Nov 2;24(23):13876–98. doi: 10.1111/jcmm.15979 (PMC7753997; doi:10.1111/jcmm.15979)
Supplement: Supplementary file 26 — Fig S7Legend [file JCMM-24-13876-s026.docx]

Figure S7 Cluster of Experimental Protein Network
