# Supplementary material for: Integrating systematic biological and proteomics strategies to explore the pharmacological mechanism of danshen yin modified on atherosclerosis
Source: J Cell Mol Med. 2020 Nov 2;24(23):13876–98. doi: 10.1111/jcmm.15979 (PMC7753997; doi:10.1111/jcmm.15979)
Supplement: Supplementary file 27 — Fig S8Legend [file JCMM-24-13876-s027.docx]

Figure S8 Cluster of Experimental Protein-Other Proteins’ PPI Network (Pink and blue circles stand for experimental protein and other protein, respectively.)
